# Costs of Single Maintenance and Reliever Therapy vs Traditional Therapy for Asthma

**DOI:** 10.1001/jamanetworkopen.2025.56757

**Published:** 2026-02-02

**Authors:** Tri Pham, Abigail R. Barker, Sarah A. Eisenstein, Eliot Jost, Anna Volerman, Krutika Chauhan, Ross C. Brownson, Timothy McBride, Mark D. Huffman, Kaharu Sumino, Lynn B. Gerald, Mario Castro, Anne E. Dixon, James G. Krings

**Affiliations:** 1 Department of Medicine, Washington University in St Louis School of Medicine, St Louis, Missouri; 2 Center for Advancing Health Services, Policy, and Economics Research, Washington University in St Louis School of Public Health, St Louis, Missouri; 3 Department of Medicine, University of Chicago Biological Sciences Division, Chicago, Illinois; 4 Department of Pediatrics, University of Chicago Biological Sciences Division, Chicago, Illinois; 5 Division of Pulmonary and Critical Care Medicine, Washington University in St Louis School of Medicine, St Louis, Missouri; 6 Prevention Research Center, Washington University in St Louis School of Public Health, St Louis, Missouri; 7 Cardiovascular Division, Global Health Center, Washington University in St Louis School of Medicine, St Louis, Missouri; 8 The George Institute for Global Health, University of New South Wales, Sydney, New South Wales, Australia; 9 Department of Medicine, Office of Population Health Sciences, University of Illinois Chicago; 10 Division of Pulmonary, Critical Care, and Sleep Medicine, University of Kansas Medical Center, Kansas City; 11 Department of Medicine, University of Vermont, Burlington

## Abstract

**Importance:**

Despite strong guideline support, single maintenance and reliever therapy (SMART) for asthma is underused in the US. Limited insurance coverage of SMART-compatible inhalers remains a major barrier to its adoption.

**Objective:**

To compare the annual asthma management costs of SMART vs traditional therapy from a US health care payer perspective.

**Design, Setting, and Participants:**

This economic evaluation used a probabilistic decision-tree model with Monte Carlo simulations to compare the total asthma management costs for patients prescribed SMART vs traditional therapy, conducting analyses from September 1, 2024, to March 13, 2025. Input data were extracted through a systematic review of 6 randomized clinical trials as well as current asthma guidelines.

**Exposures:**

SMART vs traditional therapy.

**Main Outcomes and Measures:**

The main outcome was annual asthma-related costs to health care payers. Model inputs, including exacerbation rates and expected inhaler utilization, were extracted from prior randomized clinical trials. Morbidity data and medication costs were obtained from national databases and inflated to 2024 US dollars. Analyses used a 1-year time horizon and were repeated with and without quality-adjusted life-years (QALYs) considered.

**Results:**

The model includes 11 988 individuals with moderate to severe asthma who participated in the randomized clinical trials. For patients prescribed SMART, the estimated total annual cost of asthma management was $2181 (95% CI, $1606-$2939) per patient compared with $2235 (95% CI, $1595-$3267) for traditional therapy. SMART was associated with an incremental gain of 0.0006 QALYs (95% CI, 0.0003-0.0011 QALYs) per patient. SMART was less costly in 57% of simulations when QALYs were excluded, was more cost-effective in 67% of simulations when QALYs were included, and produced a mean incremental net monetary benefit of $118 (95% CI, −$344 to $663) per patient per year.

**Conclusions and Relevance:**

The findings of this economic analysis suggest that SMART was associated with modest cost savings and improved health outcomes compared with traditional asthma therapy. Given its cost-effectiveness, demonstrated effectiveness, and strong guideline endorsement, expanding insurance coverage of SMART may reduce asthma-related morbidity while lowering costs to US health care payers.

## Key Points

**Question:**

Does guideline-recommended single maintenance and reliever therapy (SMART) for asthma cost less than traditional therapy for US health care payers?

**Findings:**

In this economic evaluation using a decision-tree model and Monte Carlo simulation approach and including data from 11 988 participants, SMART was less costly than traditional therapy for US health care payers.

**Meaning:**

The findings of this study suggest that expanding coverage of SMART could provide financial benefits to health care payers.

## Teaser

This economic evaluation compares the annual asthma management costs of guideline-recommended single maintenance and reliever therapy vs traditional therapy from a US health care payer perspective.

## Introduction

Asthma is the most prevalent chronic respiratory disease worldwide.^1^ In the US, more than half of individuals with asthma experience a severe exacerbation each year,^2,3^ at substantial costs to health care payers.^4^ In 2019 and 2020, the Global Initiative for Asthma (GINA)^5^ and the National Asthma Education and Prevention Program (NAEPP)^6^ began strongly recommending inhaled corticosteroid (ICS)–formoterol as both daily maintenance therapy and rescue therapy for moderate to severe asthma. This approach—known as single maintenance and reliever therapy (SMART) or maintenance and reliever therapy—reduces the risk of severe exacerbations by approximately one-third.^7,8^

Although SMART is evidence-based and guideline-recommended,^5,6^ global use remains limited,^9,10,11^ representing a major implementation gap in asthma management.^12^ In the US, many pharmacy benefit managers (PBMs) do not place ICS-formoterol pressurized metered-dose inhalers (pMDIs)—the only SMART-compatible inhalers—on their preferred drug lists, thereby restricting patient access.^12,13^ Some formularies cover only one ICS-formoterol inhaler per month,^14^ which is often insufficient for SMART, as it requires both maintenance and reliever use.^12^ Inadequate coverage is a primary barrier to widespread adoption of SMART in the US.^15,16^

A better understanding of the comparative costs of SMART vs traditional asthma therapy could influence payer decisions. Therefore, we conducted the first (to our knowledge) US-based cost analysis comparing the estimated annual cost of asthma care for patients prescribed SMART vs traditional therapy from a health care payer perspective. We hypothesized that, despite higher medication costs, SMART would be less costly due to reduced downstream asthma-related morbidity.

## Methods

### Study and Model Overview

**Figure 1.**
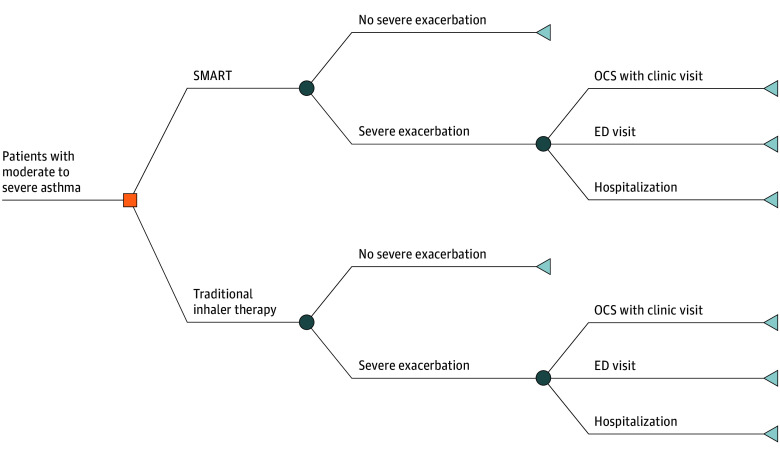
Decision-Tree Model The decision-tree model was used to evaluate the probability of potential health outcomes associated with single maintenance and reliever therapy (SMART) vs traditional inhaler therapy in moderate to severe asthma. In this model, 4 possible health states were considered, including the absence of a severe exacerbation and 3 categories of severe exacerbation: outpatient management with an oral corticosteroid (OCS) prescription and clinic visit, emergency department (ED) visit, or inpatient hospitalization for asthma. The orange square represents a decision node, where a choice between 2 treatment options is possible. The circles represent chance nodes and indicate the uncertainty between outcomes. The triangles represent end point nodes and indicate final outcomes.

For this economic analysis, which was conducted from September 1, 2024, to March 13, 2025, we developed a probabilistic decision-tree model to compare estimated inhaler utilization and asthma morbidity outcomes for patients prescribed SMART vs traditional therapy (Figure 1). Each strategy was assigned an annualized probability of 4 health states: the absence of a severe asthma exacerbation or 1 of 3 conventionally accepted severe exacerbation types: outpatient management, emergency department (ED) visit, or inpatient hospitalization. Clinical probabilities for each outcome were derived from prior randomized clinical trials (RCTs) of SMART vs traditional therapy.^17,18,19,20,21,22^ Potentially eligible RCTs were identified through an Excerpta Medica database (EMBASE) search and supplemented by manual review of references from the NAEPP’s *2020 Focused Updates to the Asthma Management Guidelines*,^6^ GINA’s 2024 *Global Strategy for Asthma Management and Prevention*,^5^ 2 Cochrane reviews,^23,24^ and 2 meta-analyses comparing SMART with traditional asthma therapy^7,8^ (eMethods 1 in Supplement 1). The Washington University School of Medicine institutional review board determined that this study did not constitute human participants research and therefore did not require institutional review board oversight or informed consent. We followed the Consolidated Health Economic Evaluation Reporting Standards (CHEERS) reporting guideline.^25^

We used a modeling approach incorporating Monte Carlo simulations over a 1-year time horizon. A Markov model was not used due to structural limitations in available RCT data^26^ (eMethods 2 in Supplement 1). Analyses were conducted with and without health-related quality of life metrics to accommodate varying health care payers’ priorities and ongoing debates regarding quality-adjusted life-years (QALYs) in payers’ decision-making.^27^

All analyses were conducted from a health care payer perspective with all costs inflated to 2024 US dollars. Final inputs were cross-checked with individual RCTs^17,18,19,20,21,22^ and meta-analyses^7,28,29^ for consistency.

### Expected Occurrence of Severe Asthma Exacerbations and Associated Costs

Annualized exacerbation rates were estimated by aggregating weighted event rates from included RCTs (eMethods 3 in Supplement 1). Exacerbation costs were derived from the 2022 Medical Expenditure Panel Survey (MEPS),^30^ and inflated to 2024 US dollars (eMethods 4 in Supplement 1). Outpatient exacerbation costs included a clinic visit and 5-day prednisone course. Data on outpatient clinical encounters that did not result in prescription of oral corticosteroids were unavailable and assumed to be equivalent between strategies. ED costs included both the cost of an ED visit and prednisone course. Hospitalization costs from the MEPS database were assumed to represent the full episode of care, including ED services provided prior to hospitalization. We did not include the costs of adverse events beyond asthma exacerbations, as rates are similar between SMART and traditional therapy.^23,24^ Long-term morbidity costs, including those from cumulative systemic corticosteroid exposure,^31^ were not considered given the 1-year time horizon.

### Expected Inhaler Actuations and Associated Costs

Annual inhaler costs were estimated by combining inhaler prices with the expected number of inhaler refills for each treatment strategy. First, we calculated per-actuation costs by dividing the inhaler’s cost by the number of actuations it contains. We then multiplied this per-actuation cost by the annualized number of inhaler actuations used by patients in the included RCTs. Use was standardized to the most commonly used inhalers in the US—budesonide-formoterol pMDI for SMART and albuterol pMDI for short-acting beta-agonist (SABA)—using standard inhaler equivalency charts^32,33,34,35,36^ (eMethods 5 and eTable in Supplement 1).

Following recommendations from the *Second Panel on Cost Effectiveness in Health and Medicine*^37^ and prior studies,^38,39^ we used the midpoint between the Veterans Affairs Federal Supply Schedule (VAFSS)^40^ and the National Average Drug Acquisition Cost (NADAC) in our primary analyses to estimate medication costs.^41^ We repeated analyses using the average wholesale price from Merative’s Micromedex (Merative US)^42^ and GoodRx prices (GoodRx Intermediate Holdings LLC)^43^ (eMethods 4 in Supplement 1).

GoodRx prices represent a patient cash-price perspective and are included only to illustrate the range of potential prices. Medication costs were extracted on October 22, 2024, and reflect prices without inclusion of pharmacy dispensing fees.

### Utility Values of Health States

After analyses were completed without QALYs, we repeated them incorporating QALYs. For periods without an exacerbation, we applied a baseline utility for moderate to severe asthma using previously published data^44^ and assumed equal baseline utilities for patients prescribed SMART and traditional asthma therapy (eMethods 6 in Supplement 1). Severe asthma exacerbations received disutility values of 0.10 (outpatient treatment with oral corticosteroids), 0.15 (ED visit), and 0.20 (hospitalization), each resulting in 2 weeks of disutility, consistent with prior studies.^45,46,47^ QALYs were not discounted due to the 1-year time horizon.^48^ We used a willingness-to-pay threshold of $100 000 per QALY.^49^

### Statistical Analysis

Our primary analysis compared annualized asthma management costs to payers when SMART vs traditional therapy was used. We conducted probabilistic sensitivity analyses with 50 000 Monte Carlo simulations, in which model inputs were varied across their plausible ranges to reflect uncertainty. The resulting percentage of simulations represents the proportion in which SMART was cost-saving or cost-effective. Scenario analyses were performed by repeating the main analyses using Micromedex and GoodRx pricing. One-way sensitivity analyses varied inhaler use, exacerbation rates, and cost parameters. Aside from MEPS-derived costs, which had available variability data, parameters were assumed to have 15% variation^50^ with gamma distributions for cost and morbidity parameters and beta distributions for utilities.^50^ All analyses were conducted in R, version 4.4.2 (R Project for Statistical Computing).

## Results

We reviewed 6249 unique citations for potential data extraction. Of these, 6127 did not meet inclusion criteria based on abstract review. A full-text review was conducted on the remaining 122 articles; 6 RCTs,^17,18,19,20,21,22^ which cumulatively analyzed 11 988 participants with moderate to severe asthma (defined as GINA steps 3-5), were included in the final model (Table 1; eFigure 1 in Supplement 1). Aggregated exacerbation rates and inhaler utilization inputs are provided in eResults 1 in Supplement 1, and model input parameters are shown in Table 2.^17,18,19,20,21,22,29,30,40,41,42,43,44,47,51^

**Table 1.** Summary of Studies Included for Input^a^

Trial and treatment	No. of participants	Exacerbation rate by type, event per patient-year^b^			Inhaler utilization, mean actuations per day^c^	
		OCS with clinic visits	ED visits	Hospitalizations	Budesonide-formoterol	Albuterol
AHEAD (2007)^18^						
SMART: budesonide-formoterol^d^	1151	0.16	0.09	0.02	4.88	NA
Control: fluticasone-salmeterol + terbutaline^d^	1153	0.18	0.10	0.02	3.96	2.02
COMPASS (2007)^19^						
SMART: budesonide-formoterol^d^	1107	0.14	0.13	0.03	3.25	NA
Control: fluticasone-salmeterol + terbutaline^d^^,^^e^	1123	0.22	0.18	0.04	4.30	2.07
Control: budesonide-formoterol + terbutaline^d^	1105	0.22	0.12	0.02	4.30	2.24
Papi et al (2013)^20^						
SMART: beclomethasone-formoterol^e^	852	0.15	0.06	0.01	2.01	NA
Control: beclomethasone-formoterol + salbutamol^e^	849	0.22	0.09	0.02	1.96	1.40
Patel et al (2013)^21^						
SMART: budesonide-formoterol^e^	151	0.38	0.10	0.05	4.72	NA
Control: budesonide-formoterol + salbutamol^e^	152	0.79	0.15	0.03	3.42	NA^f^
SAKURA (2013)^17^						
SMART: budesonide-formoterol^d^	1049	0.15	0.13	0.01	3.21	NA
Control: budesonide-formoterol + terbutaline^d^	1042	0.21	0.21	0.03	2.00	2.92
SMILE (2006)^22^						
SMART: budesonide-formoterol^d^	1113	0.15	0.04	0.01	2.98	NA
Control: budesonide-formoterol + terbutaline^d^	1141	0.30	0.07	0.01	1.99	2.52

Abbreviations: AHEAD, Comparison of the Efficacy/Safety of Symbicort Turbuhaler, Seretide Diskus 50/500 µg & Terbutaline Turbuhaler 0.4 mg; COMPASS, Comparison vs Higher Fixed-Dose of Symbicort and Seretide; ED, emergency department; NA, not applicable; OCS, oral corticosteroids; pMDI, pressurized metered-dose inhaler; SAKURA, Study to Investigate the Efficacy of Symbicort SMART; SMART, single maintenance and reliever therapy; SMILE, Effect of Budesonide in Combination With Eformoterol for Reliever Therapy in Asthma Exacerbations: a Randomised Controlled, Double-Blind Study.

^a^ Six studies met the inclusion criteria and were then aggregated and weighted based on patient-years for model input. Because individual studies did not consistently report variance for outcomes or actuations, we derived an overall variance from the weighted pooled means, as detailed in eMethods 3 and 5 in Supplement 1.

^b^ Severe exacerbations were categorized as events requiring at least 1 of the following: OCS for 3 days or more, ED visit, or inpatient hospitalization.

^c^ Actuations are expressed in budesonide-formoterol and albuterol pMDI equivalents. Refer to eMethods 5 in Supplement 1 for further details on conversions.

^d^ Dry powder inhaler.

^e^ Pressurized metered-dose inhaler.

^f^ The study by Patel et al^21^ did not provide data on short-acting beta-agonist actuation frequency.

**Table 2.** Model Inputs

Model input^a^	Value	Distribution	Source
Exacerbation rate, mean events per patient-year (95% CI)^b^			
OCS with clinic visit			
SMART	0.15 (0.13-0.18)	Gamma	Bousquet et al,^18^ 2007; Kuna et al,^19^ 2007; Papi et al,^20^ 2013; Patel et al,^21^ 2013; Rabe et al,^22^ 2006
Traditional therapy	0.24 (0.21-0.28)		
ED visit			
SMART	0.09 (0.08-0.10)	Gamma	Atienza et al,^17^ 2013; Bousquet et al,^18^ 2007; Kuna et al,^19^ 2007; Papi et al,^20^ 2013; Patel et al,^21^ 2013; Rabe et al,^22^ 2006; Wickstrøm et al,^29^ 2009
Traditional therapy	0.13 (0.11-0.15)		
Inpatient hospitalization			
SMART	0.01 (0.01-0.02)	Gamma	Atienza et al,^17^ 2013; Bousquet et al,^18^ 2007; Kuna et al,^19^ 2007; Papi et al,^20^ 2013; Patel et al,^21^ 2013; Rabe et al,^22^ 2006; Wickstrøm et al,^29^ 2009; Arrotta et al,^51^ 2019
Traditional therapy	0.02 (0.02-0.03)		
Inhaler utilization, mean actuations per day (95% CI)			
Budesonide-formoterol 160 µg-4.5 µg, 120 actuations			
SMART	3.31 (2.83-3.82)	Gamma	Atienza et al,^17^ 2013; Bousquet et al,^18^ 2007; Kuna et al,^19^ 2007; Papi et al,^20^ 2013; Patel et al,^21^ 2013; Rabe et al,^22^ 2006
Traditional therapy	2.76 (2.36-3.19)		
Albuterol 90 µg, 200 actuations			
SMART	NA	Gamma	Atienza et al,^17^ 2013; Bousquet et al,^18^ 2007; Kuna et al,^19^ 2007; Papi et al,^20^ 2013; Patel et al,^21^ 2013; Rabe et al,^22^ 2006
Traditional therapy	2.27 (1.95-2.63)		
Exacerbation costs, $ (95% CI)			
OCS with clinic visit	236.11 (2.58-1365.05)	Gamma	Medical Expenditure Panel Survey^30^
ED visit	825.79 (28.69-2950.97)	Gamma	Medical Expenditure Panel Survey^30^
Inpatient hospitalization	13 999.64 (315.32-52 457.65)	Gamma	Medical Expenditure Panel Survey^30^
Inhaler cost, $ (95% CI)			
Budesonide-formoterol 160 µg-4.5 µg, 120 actuations			
VAFSS-NADAC	186.45 (146.99-230.53)	Gamma	OPAL,^40^ 2025; NADAC,^41^ 2024
GoodRx	97.09 (73.48-123.72)	Gamma	GoodRx^43^
AWP	275.98 (200.89-362.86)	Gamma	Micromedex^42^
Albuterol 90 µg, 200 actuations			
VAFSS-NADAC	41.11 (27.25-57.78)	Gamma	OPAL,^40^ 2025; NADAC,^41^ 2024
GoodRx	25.54 (19.21-32.77)	Gamma	GoodRx^43^
AWP	68.14 (49.49-89.40)	Gamma	Micromedex^42^
Utilities, QALY (95% CI)			
Baseline utility	0.73 (0.62-0.83)	Beta	Oh et al^44^
Disutility			
OCS with clinic visit	0.10 (0.09-0.12)	Beta	Lloyd et al,^47^ 2007
ED visit	0.15 (0.13-0.17)	Beta	Lloyd et al,^47^ 2007
Inpatient hospitalization	0.20 (0.17-0.23)	Beta	Lloyd et al,^47^ 2007

Abbreviations: AWP, average wholesale price; ED, emergency department; NA, not applicable; NADAC, National Average Drug Acquisition Cost; OCS, oral corticosteroids; OPAL, Office of Procurement, Acquistion, and Logistics; QALY, quality-adjusted life-year; SMART, single maintenance and reliever therapy; VAFSS, Veterans Affairs Federal Supply Schedule.

^a^ Outpatient exacerbation costs were inclusive of an acute clinic visit and a 5-day course of prednisone, 40 mg/d. ED exacerbation costs were inclusive of costs for the ED visit and a 5-day course of prednisone. Hospitalization costs included both costs of the ED visit and the inpatient stay.

^b^ Severe exacerbations were categorized as events requiring at least 1 of the following: OCS for 3 days or more, ED visit, or inpatient hospitalization.

### Main Analysis Using VAFSS-NADAC Pricing

SMART resulted in cost savings for health care payers in 57% of simulations. The mean annual cost of asthma management was $2181 (95% CI, $1606-$2939) per patient for SMART, compared with $2235 (95% CI, $1595-$3267) for traditional therapy. Inhaler costs accounted for approximately 80% of the direct asthma management costs in both groups. The mean annual inhaler cost was $1877 (95% CI, $1410-$2424) per patient for SMART vs $1738 (95% CI, $1345-$2025) for traditional therapy. However, the annual asthma-related morbidity costs were lower for SMART, at $304 (95% CI, $6-$1383) per patient compared with $497 (95% CI, $10-$2256) per patient for traditional therapy. Analyses using VAFSS and NADAC pricing independently are shown in eResults 2 in Supplement 1.

### Scenario Analyses Using Alternative Inhaler Pricing

**Figure 2.**
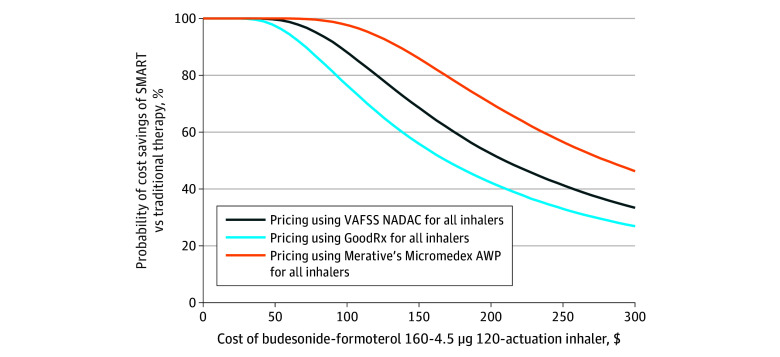
Probability of Cost Savings of Single Maintenance and Reliever Therapy (SMART) vs Traditional Inhaler Therapy Over a Range of Budesonide-Formoterol Inhaler Costs Using 3 Pricing Databases The price point for each of the 3 inhaler cost databases for budesonide-formoterol, 160-4.5 µg, and albuterol, 90 µg, is shown as of October 22, 2024, with the probability that SMART is cost saving and the mean cost savings per patient-year immediately below. AWP indicates average wholesale price; VAFSS NADAC, Veterans Affairs Federal Supply Schedule National Average Drug Acquisition Cost.

We repeated analyses using alternative sources for inhaler pricing. Using Micromedex’s average wholesale price, SMART was cost saving in 52% of simulations, with annual costs of $3084 (95% CI, $2176-$4207) per patient for SMART vs $3100 (95% CI, $2208-$4328) for traditional therapy. Using GoodRx pricing, SMART was cost saving in 78% of simulations, with a mean annual cost of $1282 (95% CI, $872-$1909) per patient for SMART vs $1419 (95% CI, $897-$2416) for traditional asthma therapy. Estimated annual savings ranged from $17 to $138 per patient depending on inhaler pricing. Cost differences across budesonide-formoterol prices are shown in Figure 2, which demonstrate that the likelihood of SMART being cost saving increases as budesonide-formoterol prices decrease. At the baseline budesonide-formoterol price of $187 per inhaler, SMART yielded $54 in annual savings per patient; reducing the price of budesonide-formoterol to $150 increased the savings to $120 per patient per year.

#### One-Way Sensitivity Analyses

**Figure 3.**
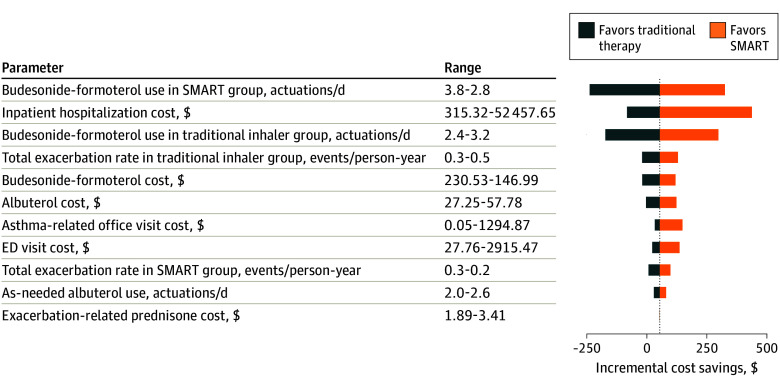
Tornado Diagram of 1-Way Sensitivity Analyses for Relevant Parameters at Veterans Affair Federal Supply Schedule (VAFSS)–National Average Drug Acquisition Cost (NADAC) Inhaler Pricing The vertical line represents the base-case scenario using 2024 VAFSS-NADAC pricing, which resulted in a mean cost savings of $54 per patient-year. ED indicates emergency department; SMART, single maintenance and reliever therapy.

One-way sensitivity analyses were conducted to evaluate how different factors were associated with cost-saving estimates. Results were most sensitive to the frequency of budesonide-formoterol actuations, severe exacerbation rates, and the cost of hospitalizations (Figure 3). Costs for ED visits and office-based asthma visits had a comparatively minor association with results. When using alternative databases that listed lower inhaler costs, the association of inhaler actuation frequency with the model’s sensitivity was reduced, suggesting that as inhaler prices decline, the frequency of inhaler use becomes less important in assessing the relative cost-effectiveness of SMART (eFigures 2 and 3 in Supplement 1).

#### Analyses With Incorporation of QALYs

When incorporating health-related quality of life metrics into the models, SMART yielded an incremental annual gain of 0.0006 QALYs (95% CI, 0.0003-0.0011 QALYs) compared with traditional asthma therapy (eResults 3 in Supplement 1). At a willingness-to-pay threshold of $100 000 per QALY, SMART was more cost-effective in 67% of simulations and produced a mean incremental net monetary benefit of $118 (95% CI, −$344 to $663) per patient per year. Acceptability curves and scatterplots illustrating the probability of SMART being cost-effective across a range of willingness-to-pay thresholds are presented in eFigures 4 and 5 in Supplement 1.

## Discussion

This US-based cost analysis of guideline-recommended SMART for asthma found that SMART was associated with reduced cumulative asthma management expenses for health care payers compared with traditional asthma treatment. Although SMART was associated with higher medication costs, reduced downstream morbidity lowered overall expenses. Estimated annual savings ranged from $17 to $138 per patient depending on inhaler pricing. Approximately 28 million individuals in the US have asthma,^52,53^ of whom 20% to 30% have moderate persistent disease^54,55,56^ and 5% to 10% have severe asthma.^56,57,58^ Even modest increases in SMART uptake could yield meaningful population-level savings. For example, increasing national SMART uptake from 10% to 20% is projected to generate approximately $250 million in direct health care cost savings over 5 years (eFigure 6 in Supplement 1).

Our findings align with prior non-US studies demonstrating that SMART is associated with reduced costs for health care payers. Studies conducted in Europe,^29,59,60^ Asia,^46^ and South America^61,62^ have shown that SMART is a cost-effective asthma management strategy. However, direct comparisons between these studies and our results are challenging due to structural and regulatory differences between countries’ health care systems. Unlike countries with centralized health care systems and stronger governmental regulation,^63^ the US operates a predominantly privatized health care model with fewer price controls,^63^ often resulting in higher medication prices^64^ and greater variability in patient access to recommended therapies. These factors underscore the importance of conducting US-specific cost analyses to guide payers’ decisions.

In our model, inhalers accounted for over 80% of the direct asthma management costs, making budesonide-formoterol inhaler pricing pivotal in cost analyses of SMART. As budesonide-formoterol prices decreased, the savings associated with SMART increased. At a baseline price of $187 per budesonide-formoterol inhaler, SMART yielded $54 in annual savings per patient; reducing the price of budesonide-formoterol to $150 increased the savings to $120 per patient per year. This finding is especially relevant given the recent entry of generic budesonide-formoterol into the market,^65^ which may further reduce prices.^66,67^

Cost estimates in our model were highly sensitive to inhaler actuation frequency, with more frequent use increasing expenses. This finding also has important cost implications for payers. Asthma guidelines recommend stepping down inhaler therapy to the lowest dose that maintains asthma control, but step-down strategies fundamentally differ between SMART and traditional therapy.^5,68^ In traditional therapy, stepping down generally involves reducing the maintenance inhaler dose while maintaining the same number of daily actuations. However, with SMART, stepping down involves reducing the number of daily maintenance inhaler actuations a patient is instructed to take with the same-dose inhaler. Given the sensitivity to actuation frequency in our model, stepping down SMART could lower costs to payers by extending inhaler refill intervals.

Adherence to maintenance inhaler therapy is lower than observed in clinical trials.^69,70,71,72^ Because lower adherence increases exacerbation risk,^73^ cost outcomes may differ from those estimated using RCT inputs (eFigure 7 in Supplement 1 models lower maintenance inhaler adherence). SMART uses the same inhaler for both maintenance and relief, meaning patients who miss scheduled maintenance doses still receive ICS during symptom worsening. This built-in linkage of an ICS to each reliever actuation has been highlighted by GINA and the NAEPP as a strategy that may partially mitigate the negative clinical consequences of maintenance nonadherence.^5,6^ Accordingly, because our model relied on RCT data with higher maintenance adherence, SMART may be even comparatively more cost-effective for payers in practice than these estimates suggest.

The incremental QALY gain for SMART in this study is modest, which may reflect the 1-year time horizon and the relatively small measurable changes in health-related quality of life over short intervals in chronic asthma. In practice, SMART could confer additional quality of life benefits through greater treatment simplicity, although robust comparative quality of life data are lacking. Longer-term analyses incorporating morbidity, exacerbation patterns, and quality of life impacts would help fully quantify SMART’s long-term value relative to traditional therapy.

We evaluated direct costs from a health care payer perspective. A societal perspective—which considers indirect costs such as productivity losses—would likely further favor the cost comparison of SMART over traditional therapy, as patients receiving SMART experience fewer exacerbations and therefore less absenteeism and reduced productivity loss. These findings would be consistent with other non-US economic evaluations of SMART that have adopted a societal perspective.^29,46,59,60^ In addition, clinician-incurred costs—such as time spent completing prior authorizations or identifying appropriate therapeutic substitutes when SMART is not covered—are not captured in this analysis.

SMART was associated with lower overall asthma management costs despite higher inhaler costs. This pattern is consistent with historical transitions in asthma care, in which higher upfront inhaler costs have produced downstream savings for payers.^74,75^ For example, before the 1990s, short-acting beta-agonist monotherapy was the standard treatment for asthma.^76^ The introduction of maintenance ICS therapy increased medication costs but ultimately reduced total health care spending by decreasing asthma-related morbidity.^77,78^ Similarly, replacing ICS-only inhalers with ICS–long-acting beta-agonist (LABA) combinations has been associated with lower health care costs.^74,75^ In contrast, biologic therapies for asthma remain cost prohibitive for many patients, and their cost-effectiveness ratios often exceed commonly accepted willingness-to-pay thresholds.^79,80^

Given its clinical effectiveness, strong guideline endorsement, and favorable cost profile, broader formulary inclusion of budesonide-formoterol SMART is warranted. We recognize that formulary decisions are often made by PBMs, whose priorities may differ from insurers and who may not be fully aware of the downstream cost savings from reduced morbidity. However, formulary coverage remains a major barrier to SMART implementation in the US, where many PBMs cover only non–formoterol-containing ICS-LABA combinations, which are incompatible with SMART. Because formoterol is the only LABA that provides both rapid symptom relief and long-acting bronchodilation, features that are necessary for SMART,^81^ ensuring coverage of budesonide-formoterol is necessary to expand access to guideline-recommended care.

Practical SMART coverage considerations merit attention. Although our model estimated a mean use of 1 inhaler every 36 days with SMART, inhaler use varies substantially. Coverage policies should therefore allow patients to obtain multiple budesonide-formoterol inhalers each month, as recommended by GINA, to ensure access during periods of frequent reliever use and across different settings.^5^ Some Medicaid and Medicare programs have already implemented policies that cover multiple budesonide-formoterol inhalers per month to enable appropriate SMART access.^14,82^

### Limitations

This study has some limitations. We relied on RCT data rather than clinical evidence for estimates of inhaler use and morbidity.^5,6,7,8^ Our model assumed the use of maintenance budesonide-formoterol pMDIs in both treatment arms, including in traditional therapy; however, while inhaler prices vary across payers’ contracts,^83^ budesonide-formoterol is generally priced similarly to other ICS-LABAs.^42^ Many SMART trials were conducted before biologics became widely used for severe asthma.^18,19,22^ Biologics have since transformed asthma care; however, they remain prescribed to only approximately 2% of patients,^84^ and SMART continues to be recommended for patients receiving biologics.^5^

Because available data did not include the longitudinal transitions required for a Markov model, we used a decision-tree framework. This approach cannot fully represent recurrent exacerbations or complex health-state transitions. Most SMART trials used the Turbuhaler dry-powder inhaler, which is unavailable in the US. One blinded RCT,^21^ however, evaluated budesonide-formoterol pMDI and demonstrated comparable benefit. Although the pMDI lacks specific US Food and Drug Administration approval for reliever use,^85^ both GINA and the NAEPP endorse SMART with a budesonide-formoterol pMDI when the pMDI is the only option.^5,6^ Thus, despite potential device-specific pharmacokinetic differences, current evidence and guidelines support pMDI-based SMART in US practice. Although our 1-year time horizon aligns with payers’ annual budget cycles, this time frame does not capture the cumulative clinical and economic effects that accrue over multiple years. Longer-term analyses incorporating robust clinical data would be helpful to fully quantify SMART’s long-term value.

## Conclusions

This economic analysis found that SMART was generally not more expensive and, in most scenarios, was modestly less costly for US health care payers than traditional asthma therapy. Substantial gaps in guideline-recommended SMART coverage remain in the US, potentially contributing to preventable morbidity. Given SMART’s demonstrated clinical benefit and its favorable cost profile observed in this analysis, expanding coverage represents a practical, evidence-based strategy to support improved asthma outcomes at the population level.

## Notes

Supplement 1. **eMethods 1.** Identification of Trials **eMethods 2.** Model Choice **eMethods 3.** Event Probability Extraction **eMethods 4.** Cost Estimates **eMethods 5.** Inhaler Actuation Data Extraction **eMethods 6.** Utility Values **eResults 1.** Inputted Data Congruence With Prior Meta-Analyses and Individual Trial Results **eResults 2.** Scenario Analyses Utilizing VAFSS and NADAC Pricing Independently **eResults 3.** Results of Analyses When Considering Quality-Adjusted Life Years **eFigure 1.** Flow Diagram **eFigure 2.** Tornado Plot Demonstrating One-Way Sensitivity Analyses for Relevant Parameters Using GoodRx Inhaler Pricing **eFigure 3.** Tornado Plot Demonstrating the One-Way Sensitivity Analysis for Relevant Parameters at Average Wholesale Prices **eFigure 4.** Probability of Cost-Effectiveness of SMART Versus Traditional Therapy **eFigure 5.** Cost-Effectiveness Plot of SMART Versus Traditional Therapy at VAFSS-NADAC Pricing **eFigure 6.** Budget Impact Analysis of SMART Versus Traditional Inhaler Therapy **eFigure 7.** Tornado Plot Demonstrating One-Way Sensitivity Analyses Modeling Decreased Inhaler Adherence **eTable.** Conversion of Inhaler Regimens to Available US Equivalents
eReferences

Supplement 2.
Data Sharing Statement
