# Supplementary material for: Costs of Single Maintenance and Reliever Therapy vs Traditional Therapy for Asthma
Source: JAMA Netw Open. 2026 Feb 2;9(2):e2556757. doi: 10.1001/jamanetworkopen.2025.56757 (PMC12865660; doi:10.1001/jamanetworkopen.2025.56757)
Supplement: Supplement 1. — eMethods 1. Identification of Trials eMethods 2. Model Choice eMethods 3. Event Probability Extraction eMethods 4. Cost Estimates eMethods 5. Inhaler Actuation Data Extraction eMethods 6. Utility Values eResults 1. Inputted Data Congruence With Prior Meta-Analyses and Individual Trial Results eResults 2. Scenario Analyses Utilizing VAFSS and NADAC Pricing Independently eResults 3. Results of Analyses When Considering Quality-Adjusted Life Years eFigure 1. Flow Diagram eFigure 2. Tornado Plot Demonstrating One-Way Sensitivity Analyses for Relevant Parameters Using GoodRx Inhaler Pricing eFigure 3. Tornado Plot Demonstrating the One-Way Sensitivity Analysis for Relevant Parameters at Average Wholesale Prices eFigure 4. Probability of Cost-Effectiveness of SMART Versus Traditional Therapy eFigure 5. Cost-Effectiveness Plot of SMART Versus Traditional Therapy at VAFSS-NADAC Pricing eFigure 6. Budget Impact Analysis of SMART Versus Traditional Inhaler Therapy eFigure 7. Tornado Plot Demonstrating One-Way Sensitivity Analyses Modeling Decreased Inhaler Adherence eTable. Conversion of Inhaler Regimens to Available US Equivalents eReferences [file jamanetwopen-e2556757-s001.pdf]

Supplementary Online Content

Pham T, Barker AR, Eisenstein SA, et al. Costs of single maintenance and reliever therapy vs traditional therapy for asthma. *JAMA Netw Open.* 2026;9(1):e2556757. doi:10.1001/jamanetworkopen.2025.56757

eMethods 1. Identification of Trials
eMethods 2. Model Choice
eMethods 3. Event Probability Extraction
eMethods 4. Cost Estimates
eMethods 5. Inhaler Actuation Data Extraction
eMethods 6. Utility Values
eResults 1. Inputted Data Congruence With Prior Meta-Analyses and Individual Trial Results
eResults 2. Scenario Analyses Utilizing VAFSS and NADAC Pricing Independently
eResults 3. Results of Analyses When Considering Quality-Adjusted Life Years
eFigure 1. Flow Diagram
eFigure 2. Tornado Plot Demonstrating One-Way Sensitivity Analyses for Relevant Parameters Using GoodRx Inhaler Pricing
eFigure 3. Tornado Plot Demonstrating the One-Way Sensitivity Analysis for Relevant Parameters at Average Wholesale Prices
eFigure 4. Probability of Cost-Effectiveness of SMART Versus Traditional Therapy
eFigure 5. Cost-Effectiveness Plot of SMART Versus Traditional Therapy at VAFSS-NADAC Pricing
eFigure 6. Budget Impact Analysis of SMART Versus Traditional Inhaler Therapy
eFigure 7. Tornado Plot Demonstrating One-Way Sensitivity Analyses Modeling Decreased Inhaler Adherence
eTable. Conversion of Inhaler Regimens to Available US Equivalents
eReferences

This supplementary material has been provided by the authors to give readers additional information about their work.

© 2026 Pham T et al. *JAMA Network Open.*

eMethods 1. Identification of Trials

To input data on expected asthma-related morbidity and medication usage, we identified randomized-controlled trials (RCTs) of single maintenance and reliever therapy (SMART) versus traditional therapy that met all of the following criteria: (1) enrolled participants ≥12 years with moderate-to-severe asthma (Global Initiative for Asthma [GINA] Steps 3-5); (2) compared the efficacy of SMART (inhaled corticosteroid [ICS]-formoterol as maintenance and reliever therapy) versus traditional therapy (ICS-long-acting beta agonist [LABA] as maintenance therapy and short-acting beta agonist [SABA] as reliever); (3) followed participants for ≥24 weeks; (4) reported asthma-related morbidity outcomes of interest (exacerbation requiring oral corticosteroids [OCS], emergency department [ED] visit, and/or inpatient hospitalization); (5) described inhaler types, dosages, and actuation frequency; and (6) did not utilize a dose titration protocol or alter inhaler dosing during the study.

We identified these potential RCTs using a systematic search of the Excerpta Medica (EMBASE) database on October 1, 2024 with a strategy adapted from Sobieraj et al.[1] The following search parameters were utilized: ('asthma'/exp OR 'asthma' OR 'wheezing'/exp OR 'wheezing' OR 'reactive airway disease'/exp OR 'reactive airway disease' OR 'bronchospasm'/exp OR 'bronchospasm' OR 'bronchoconstriction'/exp OR 'bronchoconstriction') AND ('fluticasone'/exp OR 'fluticasone' OR 'ciclesonide'/exp OR 'ciclesonide' OR 'budesonide'/exp OR 'budesonide' OR 'flunisolide'/exp OR 'flunisolide' OR 'beclometasone'/exp OR 'beclometasone' OR 'mometasone'/exp OR 'mometasone' OR 'triamcinolone'/exp OR 'triamcinolone' OR 'corticosteroid'/exp OR 'corticosteroid' OR 'single maintenance and reliever therapy'/exp OR 'single maintenance and reliever

© 2026 Pham T et al. *JAMA Network Open.*

therapy') AND ('formoterol'/exp OR 'formoterol'). Notably, we excluded the COSMOS study,[2] an RCT comparing SMART to traditional therapy, from our analyses due to its dose-titration design.

© 2026 Pham T et al. *JAMA Network Open.*

eMethods 2. Model Choice

Our decision to employ a probabilistic simulation model with a Monte Carlo simulation approach, instead of a dynamic Markov model, was based on careful consideration of our dataset, research question, study objectives, and the strength and limitations of each model type. While Markov models are well-suited for analyzing chronic conditions with ongoing transitions between health states, our study utilized aggregated data from clinical trials reporting static probabilities without time-dependent state transitions. Markov models applied to such highly aggregated data may introduce bias due to imprecise or unsupported transition estimates.[3] Further, a static simulation framework aligns better with our study's shorter one-year time horizon and avoids the potential issues with modeling transition probabilities, which demands more granular datasets, and if not properly met, can lead to significant bias.[3,4] Additionally, the one-year time horizon, aligned with typical health insurance cycles, further justified this modeling approach. Finally, we believed that the relative simplicity of the probabilistic simulation model, especially when visually represented in a decision-tree format, may be easier for healthcare payors to interpret.[5]

eMethods 3. Event Probability Extraction

We extracted the probability of asthma exacerbations by type (i.e., clinic visit with oral corticosteroids [OCS] prescribed, emergency department [ED] visit for asthma, and inpatient hospitalization for asthma) directly from the selected RCTs. We then aggregated and weighted these data based on the number of enrolled participants and treatment duration in each study. Some studies reported composite outcomes that combined ED visits and hospitalizations, which did not align with our model's requirement for separate rates of each exacerbation event. To address this limitation, we leveraged data from a Denmark-based cost-effectiveness analysis[6] funded by AstraZeneca (Cambridge, United Kingdom), the same sponsor as many of the selected RCTs. This analysis utilized the primary data from these RCTs and provided rates of exacerbation type, even though such breakdowns were not included in the original manuscripts. When specific hospitalization rates were not provided, we estimated that 20% of the ED visit rate would result in a hospitalization based on prior literature.[7] For studies that reported both the annual rate of all exacerbations and combined ED/hospitalization events but did not directly provide OCS exacerbation rates, we calculated the OCS rate by subtracting the combined ED/hospitalization rate from the total exacerbation rate.

© 2026 Pham T et al. *JAMA Network Open.*

eMethods 4. Cost Estimates

For exacerbation costs, we utilized data from the Agency for Healthcare Research and Quality's (AHRQ) 2022 Medical Expenditure Panel Survey (MEPS).[8] MEPS provides information on the utilization of medical services and their associated costs for the non-institutionalized US civilian population.[9] We extracted mean costs and their standard deviations for asthma-related office-based visits, 5 days of prednisone, asthma-related ED visits, and asthma-related inpatient hospitalizations with ED visit included from MEPS. To adjust for inflation, we applied the US Bureau of Labor Statistics Consumer Price Index for All Urban Consumers (CPI-U) table,[10] dividing the weighted mean cost by the June 2022 CPI and multiplying the result by the September 2024 CPI.

For inhaler costs, we referenced the Veterans Affairs Federal Supply Schedule (VAFSS) and the National Average Drug Acquisition Cost (NADAC) on October 22nd, 2024. VAFSS reflects the negotiated drug prices available to the US Department of Veterans Affairs from drug manufacturers, while the NADAC, which is calculated by the Centers for Medicare and Medicaid Services, represents the nationwide average of retail pharmacy acquisition costs for prescription medications. The Second Panel on cost-effectiveness in Health and Medicine[11] recommends incorporating VAFSS pricing, and prior guidelines recommend using the midpoint between VAFSS and NADAC for analysis for medication costs.[12] We systematically identified all listings of budesonide-formoterol 160-4.5μg inhalers with 120 doses and albuterol 90μg inhalers with 200 doses and calculated the mean price across these listings. The average prices for budesonide-formoterol inhalers were $164.90 (VAFSS) and $208.21 (NADAC), while for

© 2026 Pham T et al. *JAMA Network Open.*

albuterol inhalers, the prices were $35.51 (VAFSS) and $46.72 (NADAC). Utilizing the midpoint of these values, the inputted VAFSS-NADAC cost of budesonide-formoterol was $186.56, and the cost of albuterol was $41.12.

To account for real-world pricing variability for healthcare payors, we repeated analyses with alternative price points for inhaler costs. To do this, we used the average wholesale price (AWP) from Merative™'s Micromedex® (Merative US, Ann Arbor, MI, US)[13] and GoodRx® (GoodRx Intermediate Holdings, LLC, Santa Monica, CA, US)[14] AWP may represent the upper bound healthcare payors are expected to pay for an inhaler as it reflects the list price set by drug wholesalers or manufacturers, although this price is frequently discounted for payors.[15,16] In contrast, GoodRx pricing may represent the lower bound as it factors in significant competitive pharmacy benefit manager (PBM) discounts.[17] On October 22nd, 2024, we accessed the Red Book® on the Micromedex® database to extract the AWP for the budesonide-formoterol 160-4.5µg ($275.96) and 18g Ventolin HFA 90µg ($68.09) inhalers. For GoodRx® estimates, we specifically searched for the lowest available prices of budesonide-formoterol and albuterol on October 22nd, 2024, with the location set to St. Louis, Missouri, United States to approximate the lowest reasonable cost to payors. The identified prices were $97.09 for budesonide-formoterol and $25.54 for albuterol.

© 2026 Pham T et al. *JAMA Network Open.*

eMethods 5. Inhaler Actuation Data Extraction

We obtained actuation frequency data directly from the selected RCTs, when available, and aggregated and weighted inputted values based on the number of enrolled participants and treatment duration of each study. For studies that did not explicitly report inhaler actuation frequency, we estimated the mean daily number of actuations using the reported mean ICS dose per unit time, adjusting for the prescribed ICS dose. In cases where neither actuation frequency nor mean ICS dose was available, we referred to the previously mentioned Denmark-based cost-effectiveness study,[6] which provided further detail on inhaler use patterns from the primary RCTs. In the single instance in which SABA actuation frequency could not be derived or reliably imputed—specifically, the Patel et al. trial[18]—we excluded that study from the pooled SABA frequency calculation to avoid introducing unverifiable assumptions. All other reported outcomes from Patel trial were retained and included in pooled analyses. The calculation of weighted standard deviations followed the same approach described in **eMethods 3**.

Given that the RCTs were conducted in diverse international settings with variations in inhaler types and dosages, we standardized all treatments to the US-available budesonide-formoterol 160-4.5μg pressurized metered-dose inhalers (pMDIs) and albuterol 90μg pMDIs utilizing equivalency charts from the American Lung Association (ALA)[19] and prior literature.[20-24] We considered budesonide-formoterol pMDI and dry powder inhalers (DPIs) to have therapeutic equivalence.[25-28] The results of these conversions can be found in **Table 2**. Additionally, given that US clinical guidelines generally recommend two puffs of SABA as needed, in contrast to some

© 2026 Pham T et al. *JAMA Network Open.*

studies that recommended only one puff of terbutaline or salbutamol for reliever use, we adjusted the reported SABA actuation frequencies by doubling actuation frequency as needed to better align with US inhaler use patterns. We calculated annualized costs from inhalers by multiplying per-actuation costs by daily actuation frequency and 365 days. Consequently, the resulting cost may not be whole intervals of inhaler prices, implying partial inhaler use—an artifact of the calculation, as inhalers are dispensed in whole units only.

© 2026 Pham T et al. *JAMA Network Open.*

eMethods 6. Utility Values

We applied a baseline utility value corresponding to partly controlled asthma, derived from Oh et al.[29] This utility estimate was obtained using the European Quality of Life 5 Dimensions 3 Level Version (EQ-5D-3L), a validated instrument for calculating quality-adjusted life years (QALY). Because the pivotal SMART and traditional therapy trials included patients with moderate-to-severe asthma (who are unlikely to have perfectly controlled symptoms), this value best reflects the baseline health status of the modeled population.

We made the assumption that patients prescribed SMART and traditional inhaler therapy had the same baseline utility when not in the state of a severe asthma exacerbation, partly due to the paucity of health-related quality of life (HRQoL) data in patients treated with SMART versus traditional inhaler therapy. The EQ-5D-3L was not administered in any RCT comparing SMART to traditional therapy to our knowledge. In part, this may be due to concerns about its sensitivity to meaningful changes in asthma symptoms, as it lacks asthma-specific domains.[30]

Asthma-specific instruments such as the Asthma Control Questionnaire (ACQ) and the Asthma Quality of Life Questionnaire (AQLQ) were collected in some studies and describe asthma control and asthma-associated HRQoL, respectively. However, these measures are not easily mapped to EQ-5D-3L or other measures to determine QALYs. To our knowledge, no direct mapping from ACQ to EQ-5D-3L exists, and only one study has attempted to map AQLQ to EQ-5D-3L.[31] Among our six RCTs, only one study included AQLQ data, and showed no significant difference between treatment arms in AQLQ-delineated QoL.[32] Notably, while the ACQ may not fully capture asthma-

© 2026 Pham T et al. *JAMA Network Open.*

related HRQoL,[33] its values were generally similar across treatment groups in our RCTs.[18,34-36] Given these challenges, we elected to assume equivalent baseline utility values between the treatment groups.

For disutility estimates, we used values identified from a prior study,[37] which assigned a decrement of 0.1 for outpatient OCS course and 0.2 for hospitalization. Although explicit disutility values for ED visits were not reported, the midpoint of 0.15 has been applied in numerous prior asthma-related cost-effectiveness analyses.[38-43] While this approach has limitations, it remains a widely accepted standard in asthma cost analyses pending more robust future data.

© 2026 Pham T et al. *JAMA Network Open.*

eResults 1. Inputted Data Congruence With Prior Meta-Analyses and Individual Trial Results

After aggregation and weighting, the expected annualized severe exacerbation rate we inputted was 0.26 exacerbations per person-year (95% confidence interval [CI]: 0.22 to 0.29) for SMART and 0.39 exacerbations per person-year (95% CI: 0.33 to 0.46) for traditional therapy. These rates compare to ranges of 0.12 to 0.53 exacerbations/year for SMART-treated participants and 0.19 to 0.97 exacerbations/year for traditional-inhaler-therapy treated patients observed in individual trials.[18,32,34-36,44] In the meta-analysis by Beasley et al.,[45] the reported annual exacerbation rates were 0.31 exacerbations/year for SMART and 0.52 exacerbations/year for traditional therapy. The mean number of budesonide-formoterol actuations we inputted for those using SMART was 3.31 (95% CI: 2.83 to 3.82) actuations/day (or about ten 120-actuation budesonide-formoterol inhalers dispensed annually), which aligned with derived values of 2.01 to 4.72 actuations/day from the individual trials.[18,32,34-36,44] This was also comparable to the aggregated and weighted value of 3.04 reported by Stallberg et al.[46] and within the range of 2.86 to 4.88 observed in a prior cost analysis.[6] For traditional therapy, the mean daily budesonide-formoterol actuation was 2.76 (95% CI: 2.36 to 3.19) actuations/day (or about eight 120-actuation budesonide-formoterol inhalers dispensed annually). This was comparable to derived values of 1.96 to 3.42 actuations/day from the individual trials.[18,32,34-36,44] Additionally, the mean daily actuation of albuterol was 2.27 (95% CI: 1.95 to 2.63) actuations/day (or about four 200-actuation albuterol inhalers dispensed annually). This was consistent with derived values of 1.40 to 2.92 from actuations/day the individual trials.[18,32,34-36,44]

© 2026 Pham T et al. *JAMA Network Open.*

eResults 2. Scenario Analyses Utilizing VAFSS and NADAC Pricing Independently

Using VAFSS, SMART was cost saving in 59% of simulations. The mean cost of asthma management for patients prescribed SMART was $1,963 USD (95% CI: $1,336 to $3,422) per patient per year versus $2,030 USD (95% CI: 1,267 to $4,113) per patient per year for traditional therapy. Using NADAC, SMART provided cost savings in 55% of simulations, with a mean cost for patients prescribed SMART of $2,400 USD (95% CI: 1,686 to $3,958) per patient per year versus $2,441 USD (95% CI: 1,605 to $4,609) per patient per year for traditional asthma therapy.

© 2026 Pham T et al. *JAMA Network Open.*

eResults 3. Results of Analyses When Considering Quality-Adjusted Life Years

When incorporating HRQoL metrics into our model, SMART was associated with 0.7238 (95% CI: 0.7236 to 0.7240) QALYs, while traditional inhaler therapy was associated with 0.7232 (95% CI: 0.7228 to 0.7235), yielding an incremental annualized QALY gain of 0.0006 (95% CI: 0.0003 to 0.0011) for SMART over traditional asthma therapy.

When the disutility duration was set to 1 week, SMART was associated with 0.7244 (95% CI: 0.7242 to 0.7245) QALYs, while traditional inhaler therapy was associated with 0.7241 (95% CI: 0.7239 to 0.7242), yielding an incremental annualized QALY gain of 0.0003 (95% CI: 0.0002 to 0.0005) for SMART over traditional asthma therapy. When the disutility duration was set to 4 weeks, SMART was associated with 0.7226 (95% CI: 0.7221 to 0.7230) QALYs, while traditional inhaler therapy was associated with 0.7213 (95% CI: 0.7206 to 0.7219), yielding an incremental annualized QALY gain of 0.0013 (95% CI:0.0006 to 0.0020) for SMART over traditional asthma therapy. At a WTP threshold of $100,000 per QALY, SMART was more cost-effective than traditional asthma therapy in 62% and 76% of simulations for disutility durations of 1 week and 4 weeks, respectively, yielding a mean incremental net monetary benefit (INMB) of $84 and $184 per patient per year.

We also conducted a scenario analysis where the disutility duration was set to 1 week for outpatient exacerbations, 2 weeks for ED visits, and 4 weeks for hospitalizations. In this context, SMART was associated with 0.7240 (95% CI: 0.7238 to 0.7242) QALYs, while traditional inhaler therapy was associated with 0.7234 (95% CI: 0.7231 to 0.7237), yielding an incremental annualized QALY gain of 0.00055 (95% CI:

0.0003 to 0.0008) for SMART over traditional asthma therapy. At a WTP threshold of $100,000 per QALY, SMART was more cost-effective than traditional asthma therapy in 66% of simulations, yielding a mean INMB of $109 per patient per year.

© 2026 Pham T et al. *JAMA Network Open.*

eFigure 1. Flow Diagram

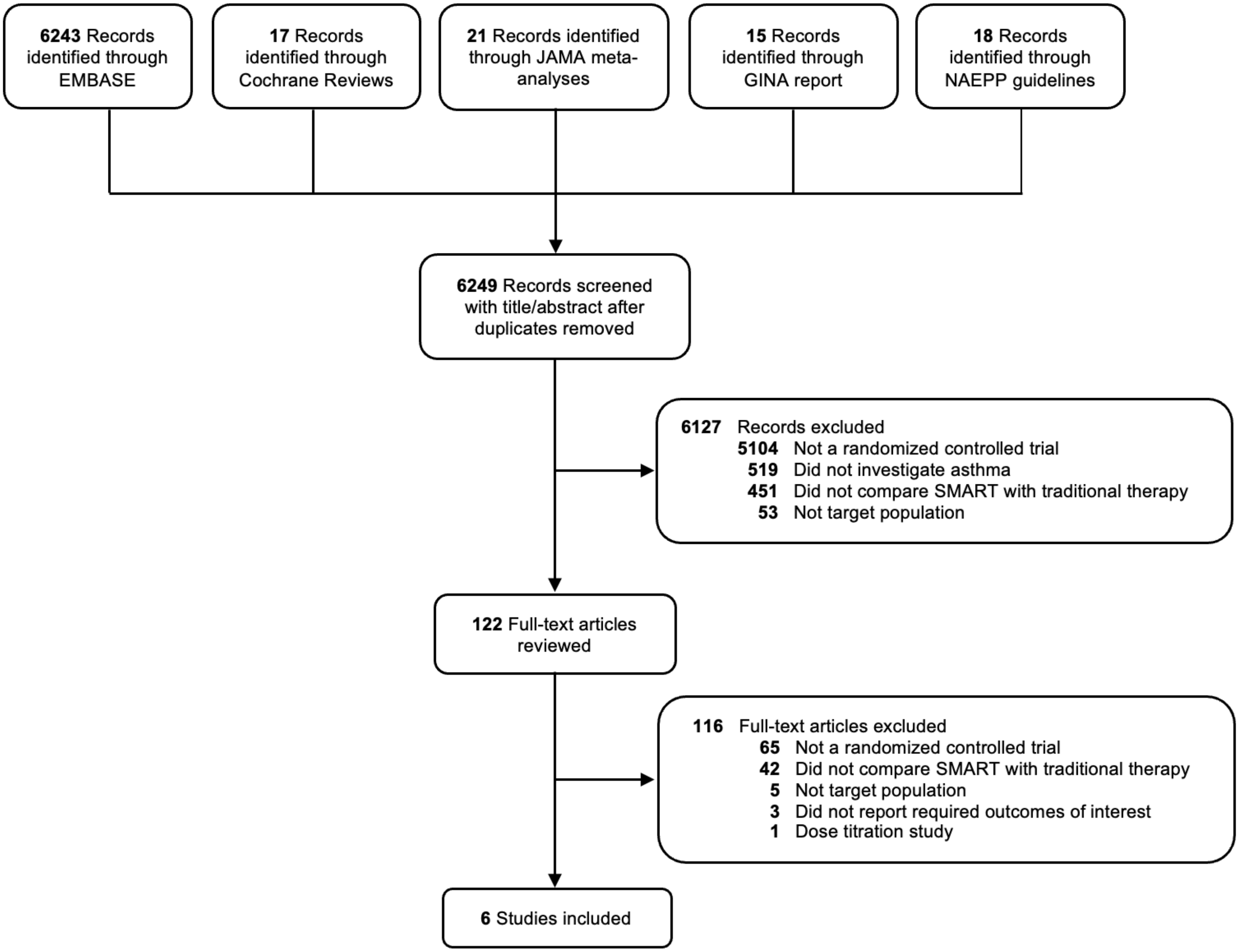

Records were reviewed step-by-step utilizing the inclusion/exclusion criteria, beginning with exclusion based on trial design and progressing iteratively.

Abbreviations used: GINA, Global Initiative for Asthma; JAMA, Journal of the American Medical Association; NAEPP, National Asthma Education and Prevention Program, SMART: single maintenance and reliever therapy.

© 2026 Pham T et al. *JAMA Network Open.*

eFigure 2. Tornado Plot Demonstrating One-Way Sensitivity Analyses for Relevant Parameters Using GoodRx Inhaler Pricing

Parameter	Lower bound	Upper bound
Inpatient hospitalization cost, USD	315.32	52457.65
Bud-form use in SMART group, actuations/day	3.82	2.83
Bud-form use in traditional inhaler group, actuations/day	2.36	3.19
Total exacerbation rate in traditional inhaler group, events/person-year	0.34	0.45
Asthma-related office visit cost, USD	0.05	1294.87
ED visit cost, USD	27.76	2915.47
Total exacerbation rate in SMART group, events/person-year	0.29	0.22
Bud-form cost, USD	123.72	73.48
Albuterol cost, USD	19.21	32.77
As-needed albuterol use, actuations/day	1.95	2.63
Exacerbation-related prednisone cost, USD	1.89	3.41

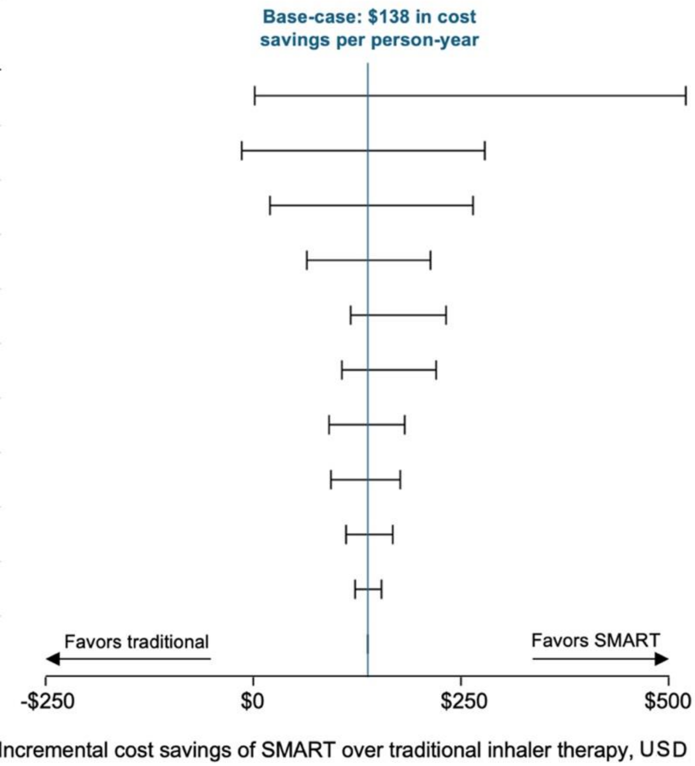

This tornado diagram depicts the one-way sensitivity analyses of various input variables on the cost saving estimates of SMART over traditional inhaler therapy. The vertical blue line represents the base-case scenario utilizing 2024 GoodRx® pricing, which resulted in a mean cost savings of $138 USD per person-year. Values above $0 (to the right) indicate cost savings favoring SMART over traditional therapy, while values below $0 (to the left) indicate cost savings favoring traditional inhaler therapy over SMART.

Abbreviations used: Bud-form, budesonide-formoterol; ED, emergency department; ICS, inhaled corticosteroids; SMART, single maintenance and reliever therapy; USD, United States dollar.

© 2026 Pham T et al. *JAMA Network Open.*

eFigure 3. Tornado Plot Demonstrating the One-Way Sensitivity Analysis for Relevant Parameters at Average Wholesale Prices

Parameter	Lower bound	Upper bound
Bud-form use in SMART group, actuations/day	3.82	2.83
Bud-form use in traditional inhaler group, actuations/day	2.36	3.19
Inpatient hospitalization cost, USD	315.32	52,457.65
Bud-form cost, USD	362.86	200.89
Albuterol cost, USD	49.49	89.40
Total exacerbation rate in traditional inhaler group, events/person-year	0.34	0.45
Asthma-related office visit cost, USD	0.05	1,294.87
ED visit cost, USD	27.76	2,915.47
Total exacerbation rate in SMART group, events/person-year	0.29	0.22
As-needed albuterol use, actuations/day	1.95	2.63
Exacerbation-related prednisone cost, USD	1.89	3.41

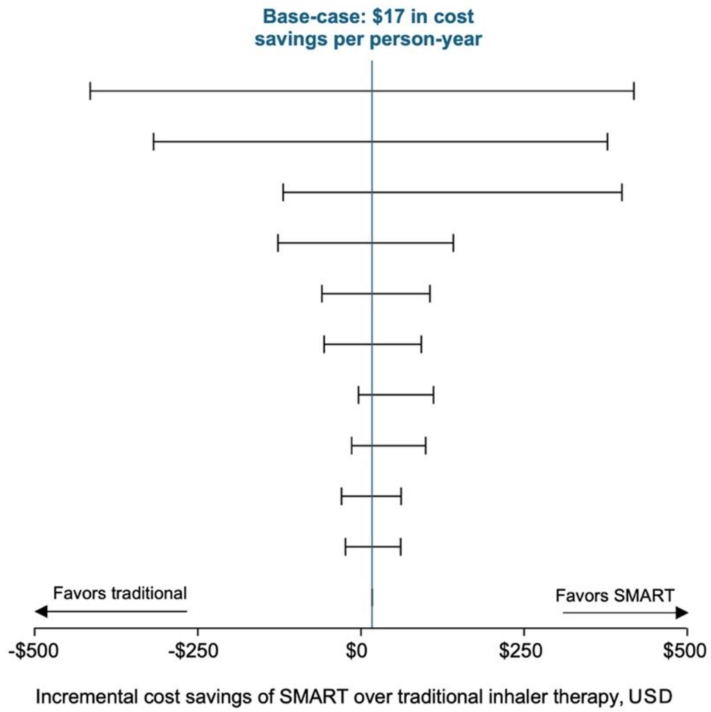

This tornado diagram depicts the one-way sensitivity analyses of various input variables on the cost saving estimates of SMART over traditional inhaler therapy. The vertical blue line represents the base-case scenario utilizing 2024 Micromedex® AWP pricing, which resulted in a mean cost savings of $17 USD per person-year. Values above $0 (to the right) indicate cost savings favoring SMART over traditional therapy, while values below $0 (to the left) indicate cost savings favoring traditional inhaler therapy over SMART.

Abbreviations used: AWP, average wholesale price; Bud-form, budesonide-formoterol; ED, emergency department; ICS, inhaled corticosteroids; SMART, single maintenance and reliever therapy; USD, United States dollar

© 2026 Pham T et al. *JAMA Network Open.*

eFigure 4. Probability of Cost-Effectiveness of SMART Versus Traditional Therapy

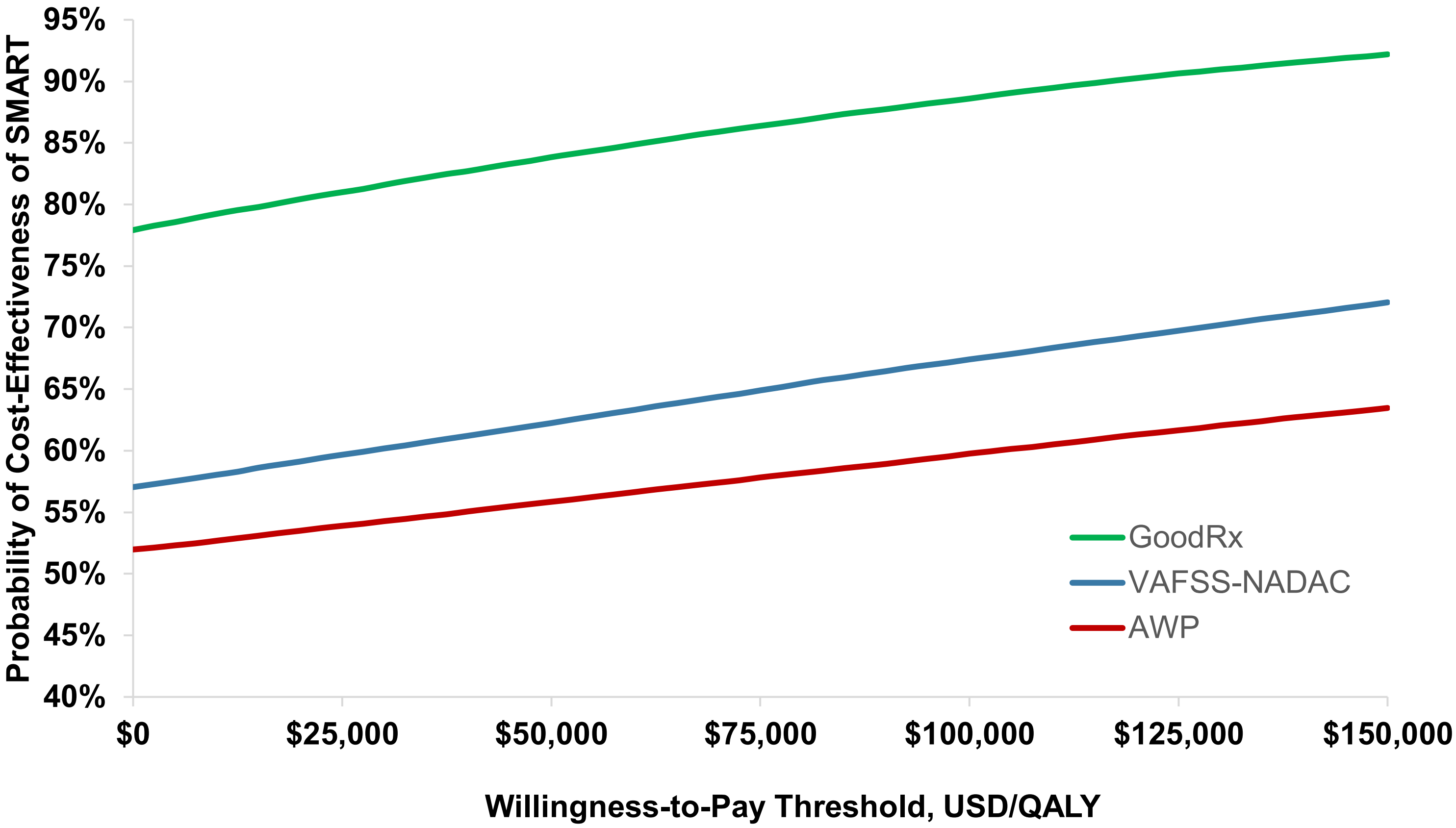

This depicts the probability of cost-effectiveness of SMART, compared to traditional inhaler therapy at varying WTP thresholds. Each colored line represents a different pricing scenario. Blue line represents the VAFSS-NADAC pricing, green line represents the GoodRx® pricing, and red line represents the AWP. The curves were generated from the results of the probabilistic analysis in which the model was run 50,000 times using Monte Carlo simulations.

Abbreviations used: AWP, average wholesale price; NADAC, National Average Drug Acquisition Cost; QALY, quality-adjusted life year; SMART, single maintenance and reliever therapy; USD, United States dollar; VAFSS, Veterans Affair Federal Supply Schedule; WTP, willingness-to-pay.

© 2026 Pham T et al. *JAMA Network Open.*

eFigure 5. Cost-Effectiveness Plot of SMART Versus Traditional Therapy at VAFSS-NADAC Pricing

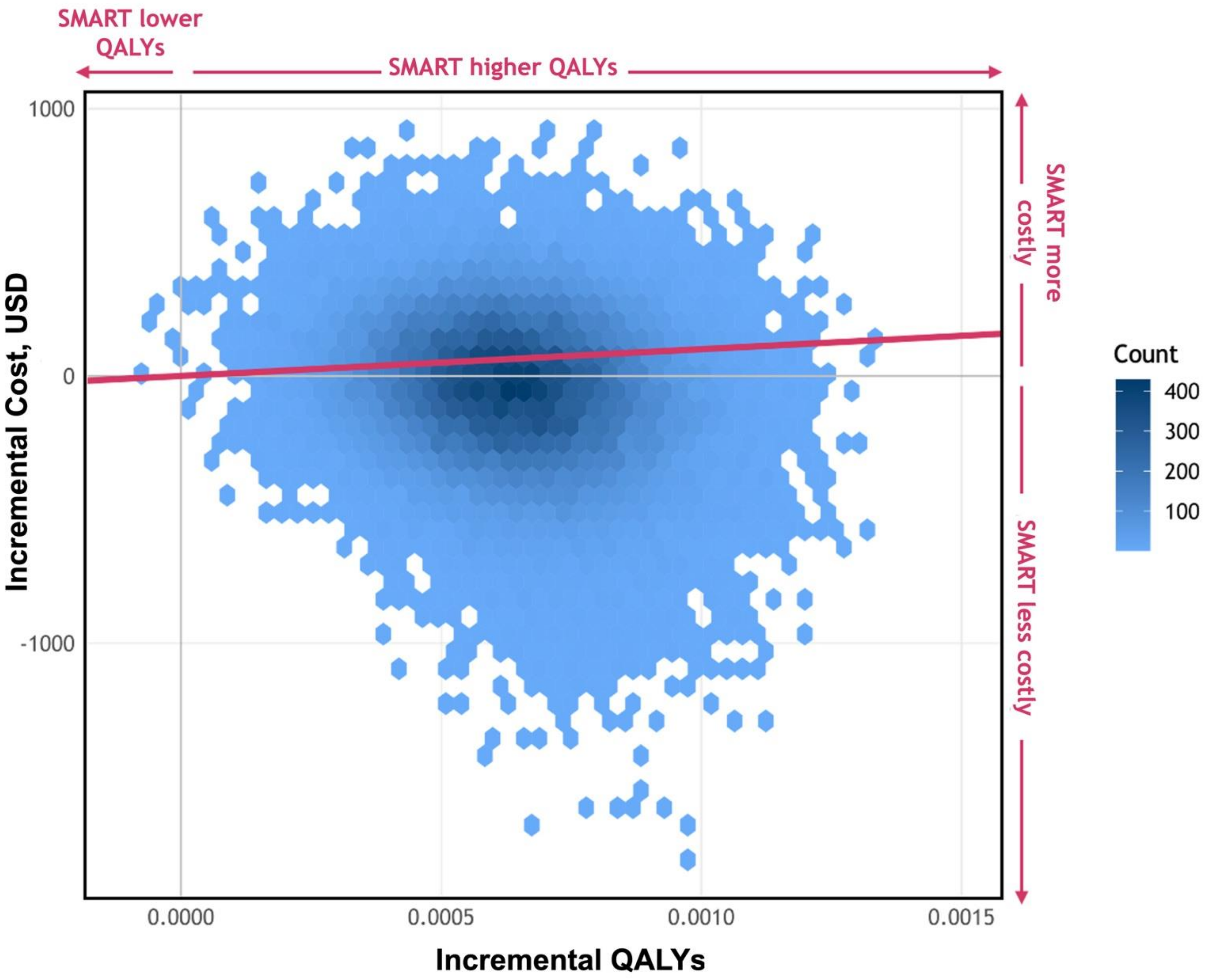

This figure shows the results of the 50,000 simulations illustrating the distribution of the incremental cost and incremental QALYs for SMART versus traditional inhaler therapy. Each hexagon represents the density of simulations, with darker blue indicating a higher concentration of simulations. The pink line denotes the willingness-to-pay threshold at $100,000 USD per QALY. Simulations below the pink line represent scenarios where SMART is most cost-effective than traditional asthma therapy, which accounts for 67% of simulations.

© 2026 Pham T et al. *JAMA Network Open.*

Abbreviations used: NADAC, National Average Drug Acquisition Cost; QALY, quality-adjusted life year; SMART, single maintenance and reliever therapy; USD, United States dollar; VAFSS, Veterans Affair Federal Supply Schedule; WTP, willingness-to-pay.

© 2026 Pham T et al. *JAMA Network Open.*

eFigure 6. Budget Impact Analysis of SMART Versus Traditional Inhaler Therapy

Budget Impact Analysis of Various SMART Uptake Scenarios

20% SMART Uptake Scenario

Treatment	Cost Type	Cost per person/year	Year 1	Year 2	Year 3	Year 4	Year 5
SMART	Inhaler Costs	$1,877	$1,708,070,000	$3,416,140,000	$5,124,210,000	$6,832,280,000	$8,540,350,000
	Outpatient Management Costs	$36	$32,760,000	$65,520,000	$98,280,000	$131,040,000	$163,800,000
	Emergency Department Costs	$74	$67,340,000	$134,680,000	$202,020,000	$269,360,000	$336,700,000
	Hospitalization Costs	$194	$176,540,000	$353,080,000	$529,620,000	$706,160,000	$882,700,000
	Total Costs	$2,181	$1,984,710,000	$3,969,420,000	$5,954,130,000	$7,938,840,000	$9,923,550,000
Traditional	Inhaler Costs	$1,738	$1,581,580,000	$3,163,160,000	$4,744,740,000	$6,326,320,000	$7,907,900,000
	Outpatient Management Costs	$57	$51,870,000	$103,740,000	$155,610,000	$207,480,000	$259,350,000
	Emergency Department Costs	$106	$96,460,000	$192,920,000	$289,380,000	$385,840,000	$482,300,000
	Hospitalization Costs	$334	$303,940,000	$607,880,000	$911,820,000	$1,215,760,000	$1,519,700,000
	Total Costs	$2,235	$2,033,850,000	$4,067,700,000	$6,101,550,000	$8,135,400,000	$10,169,250,000

30% SMART Uptake Scenario

Treatment	Cost Type	Cost per person/year	Year 1	Year 2	Year 3	Year 4	Year 5
SMART	Inhaler Costs	$1,877	$3,416,140,000	$6,832,280,000	$10,248,420,000	$13,664,560,000	$17,080,700,000
	Outpatient Management Costs	$36	$65,520,000	$131,040,000	$196,560,000	$262,080,000	$327,600,000
	Emergency Department Costs	$74	$134,680,000	$269,360,000	$404,040,000	$538,720,000	$673,400,000
	Hospitalization Costs	$194	$353,080,000	$706,160,000	$1,059,240,000	$1,412,320,000	$1,765,400,000
	Total Costs	$2,181	$3,969,420,000	$7,938,840,000	$11,908,260,000	$15,877,680,000	$19,847,100,000
Traditional	Inhaler Costs	$1,738	$3,163,160,000	$6,326,320,000	$9,489,480,000	$12,652,640,000	$15,815,800,000
	Outpatient Management Costs	$57	$103,740,000	$207,480,000	$311,220,000	$414,960,000	$518,700,000
	Emergency Department Costs	$106	$192,920,000	$385,840,000	$578,760,000	$771,680,000	$964,600,000
	Hospitalization Costs	$334	$607,880,000	$1,215,760,000	$1,823,640,000	$2,431,520,000	$3,039,400,000
	Total Costs	$2,235	$4,067,700,000	$8,135,400,000	$12,203,100,000	$16,270,800,000	$20,338,500,000

40% SMART Uptake Scenario

Treatment	Cost Type	Cost per person/year	Year 1	Year 2	Year 3	Year 4	Year 5
SMART	Inhaler Costs	$1,877	$5,124,210,000	$10,248,420,000	$15,372,630,000	$20,496,840,000	$25,621,050,000
	Outpatient Management Costs	$36	$98,280,000	$196,560,000	$294,840,000	$393,120,000	$491,400,000
	Emergency Department Costs	$74	$202,020,000	$404,040,000	$606,060,000	$808,080,000	$1,010,100,000
	Hospitalization Costs	$194	$529,620,000	$1,059,240,000	$1,588,860,000	$2,118,480,000	$2,648,100,000
	Total Costs	$2,181	$5,954,130,000	$11,908,260,000	$17,862,390,000	$23,816,520,000	$29,770,650,000
Traditional	Inhaler Costs	$1,738	$4,744,740,000	$9,489,480,000	$14,234,220,000	$18,978,960,000	$23,723,700,000
	Outpatient Management Costs	$57	$155,610,000	$311,220,000	$466,830,000	$622,440,000	$778,050,000
	Emergency Department Costs	$106	$289,380,000	$578,760,000	$868,140,000	$1,157,520,000	$1,446,900,000
	Hospitalization Costs	$334	$911,820,000	$1,823,640,000	$2,735,460,000	$3,647,280,000	$4,559,100,000
	Total Costs	$2,235	$6,101,550,000	$12,203,100,000	$18,304,650,000	$24,406,200,000	$30,507,750,000

Cost Comparison

Budget Impact Difference (20% Uptake)	-$54	-$49,140,000	-$98,280,000	-$147,420,000	-$196,560,000	-$245,700,000
Budget Impact Difference (30% Uptake)	-$54	-$98,280,000	-$196,560,000	-$294,840,000	-$393,120,000	-$491,400,000
Budget Impact Difference (40% Uptake)	-$54	-$147,420,000	-$294,840,000	-$442,260,000	-$589,680,000	-$737,100,000

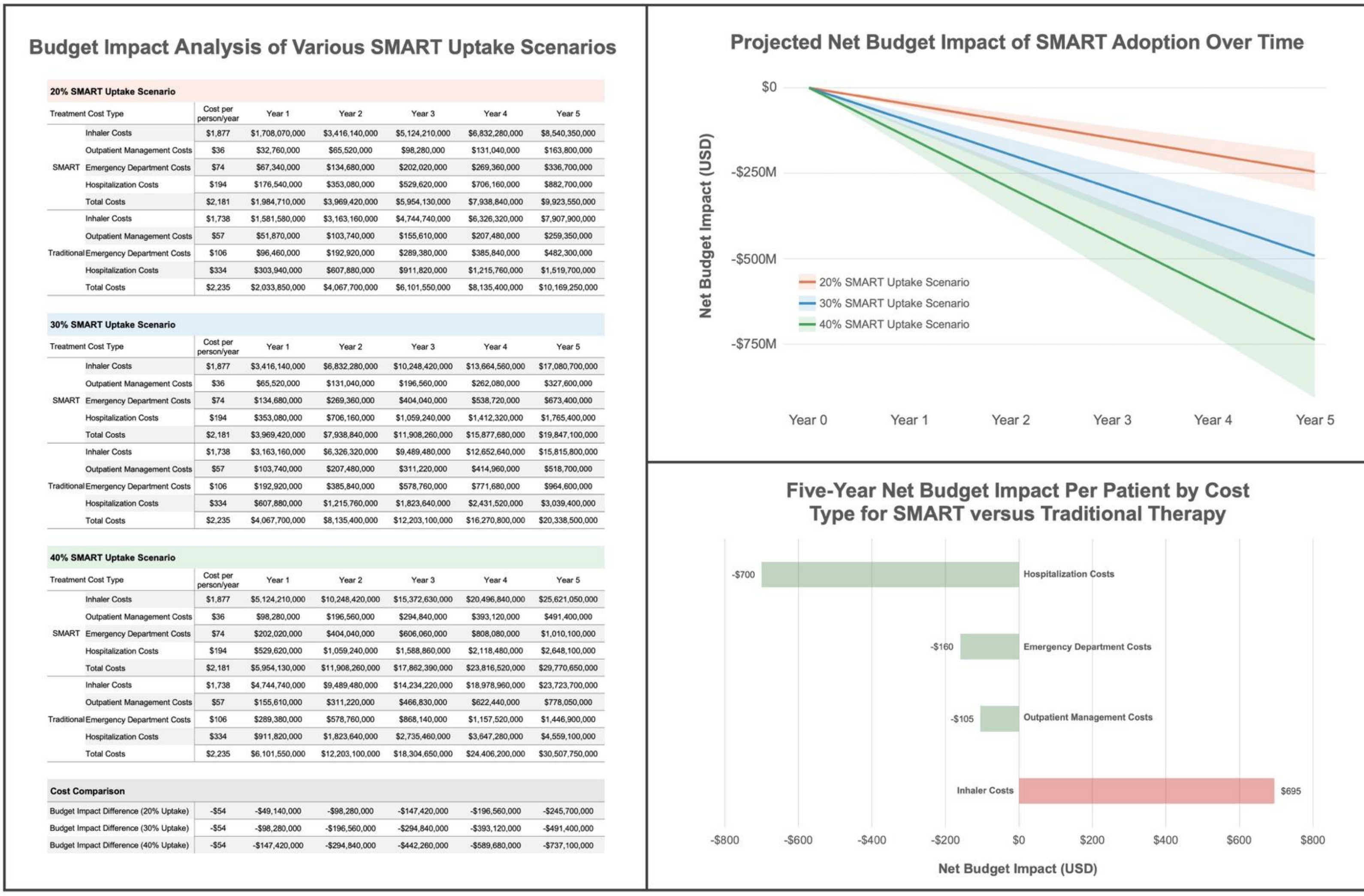

This figure presents the BIA of various SMART uptake scenarios over a five-year period based on our model. The left panel displays a table estimating the budget impact (by cost type and total cost) at 20%, 30%, and 40% SMART uptake in the US population, with the bottom portion summarizing the five-year net budget impact for each scenario. The top right panel graphically illustrates the projected net budget impact over five years, with negative values corresponding to a reduction to the budget (i.e., cost savings). The orange line, blue line, and green line represent the 20%, 30%, and 40% uptake scenario, respectively. The corresponding shaded areas represent the range of potential values based on our population estimates. The bottom right panel displays the five-year net budget impact by cost type for each person if they were on SMART versus traditional therapy. The green (negative) bars represent cost reductions, while the red (positive) bar represents cost increases to the budget.

In the US, there is an estimated 28 million individuals affected by asthma,[47,48] with 20-30% having moderate disease[49-51] and 5-10% having severe disease.[51-53] In the top

© 2026 Pham T et al. *JAMA Network Open.*

right panel, the solid bold lines represent the midpoint of the population estimates (32.5% of 28 million, or 9.1 million individuals) while the shaded areas represent the plausible range (25-40% of 28 million, or 7-11.2 million individuals). Current SMART uptake is estimated at 10%.[54] We modeled SMART uptake increases of 20%, 30%, and 40% relative to this baseline. This analysis includes only direct costs (approximately $54 net savings per person per year) and excludes indirect costs, such as productivity loss or QALY changes.

Abbreviations used: BIA, budget impact analysis; M, million; QALYs, quality-adjusted life years; SMART, single maintenance and reliever therapy; US, United States; USD, United States dollars.

© 2026 Pham T et al. *JAMA Network Open.*

eFigure 7. Tornado Plot Demonstrating One-Way Sensitivity Analyses Modeling Decreased Inhaler Adherence

Parameter	Range
Bud-form use in SMART group, actuations/day	3.82 to 0.99
Bud-form use in traditional inhaler group, actuations/day	0.83 to 3.19
Inpatient hospitalization cost, USD	315.32 to 52,457.65
Total exacerbation rate in traditional inhaler group, events/person-year	0.34 to 0.45
Bud-form cost, USD	230.53 to 146.99
Albuterol cost, USD	27.25 to 57.78
Asthma-related office visit cost, USD	0.05 to 1,294.87
ED visit cost, USD	27.76 to 2,915.47
Total exacerbation rate in SMART group, events/person-year	0.29 to 0.22
As-needed albuterol use, actuations/day	1.95 to 2.63
Exacerbation-related prednisone cost, USD	1.89 to 3.41

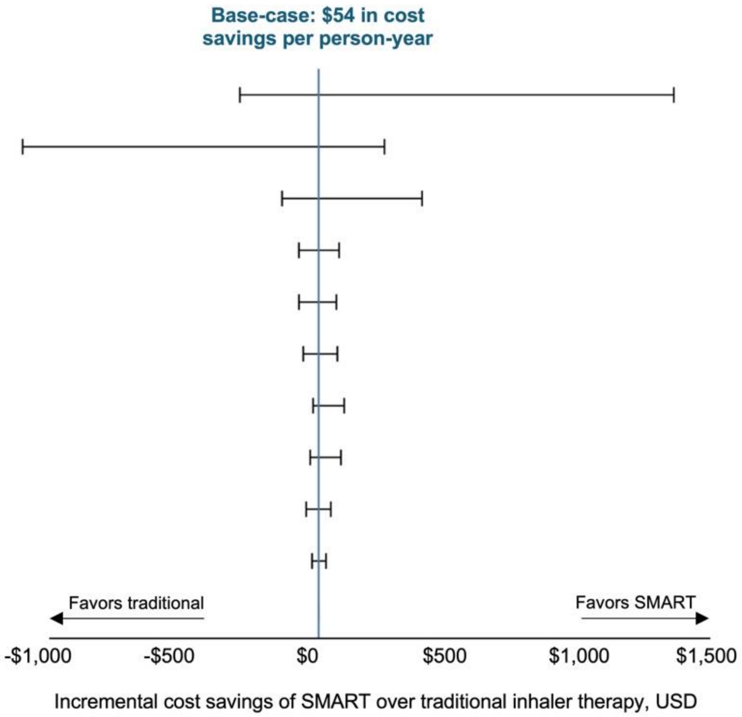

This tornado diagram depicts the one-way sensitivity analyses of various input variables on the cost saving estimates of SMART over traditional inhaler therapy using VAFSS-NADAC pricing. The vertical blue line represents the base-case scenario utilizing 2024 VAFSS-NADAC pricing, which resulted in a mean cost savings of $54 USD per person-year. Values above $0 (to the right) indicate cost savings favoring SMART over traditional therapy, while values below $0 (to the left) indicate cost savings favoring traditional inhaler therapy over SMART. This is identical to Figure 3 in the main manuscript, except that the lower bound for inhaler actuations has been extended to approximate 30% adherence, reflecting published real-world estimates of maintenance inhaler use.[55-58] This plot is intended to illustrate the relative cost outcomes under adherence levels closer to real-world practice, which are often lower than those observed in RCTs. However, while one-way sensitivity analyses inherently imply independence between variables, that is not the case in real-world circumstances. For example, reduced adherence to maintenance inhalers would be expected to increase both exacerbations and rescue inhaler use. Therefore, this figure should be interpreted with the following caveats: variables are not fully independent, changes in adherence affect multiple model parameters simultaneously, and current evidence is insufficient to accurate quantify these interdependencies.

© 2026 Pham T et al. *JAMA Network Open.*

Abbreviations used: Bud-form, budesonide-formoterol; ED, emergency department; ICS, inhaled corticosteroids; RCTs, randomized controlled trials; SMART, single maintenance and reliever therapy; NADAC, National Average Drug Acquisition Cost; USD, United States dollar; VAFSS, Veterans Affair Federal Supply Schedule.

© 2026 Pham T et al. *JAMA Network Open.*

eTable. Conversion of Inhaler Regimens to Available US Equivalents

Study dose	Trial	US equivalent regimen	Conversion reference
Bud-Form 320-9µg Turbuhaler one puff BID	COMPASS[32]	Bud-Form 160-4.5µg pMDI two puffs BID	19
Bud-Form 200-6µg DPI two puffs BID	Patel et al.[18]	Bud-Form 160-4.5µg pMDI two puffs BID	[23,24]
Bec-Form 100-6µg pMDI one puff BID	Papi et al.[35]	Bud-Form 160-4.5µg pMDI one puff BID	22
Flu-Sal 500-50µg DPI one puff BID	AHEAD[34]	Bud-Form 160-4.5µg pMDI two puffs BID	19
Flu-Sal 125-25µg pMDI/DPI two puffs BID	COMPASS[32]	Bud-Form 160-4.5µg pMDI two puffs BID	19
Salb 100µg pMDI one puff PRN	Papi et al.,[35] Patel et al.[18]	Albuterol 90µg pMDI two puffs PRN	[20,21]
Terb 0.4mg pMDI/DPI one puff PRN	COMPASS,[32] AHEAD,[34] SMILE,[36] SAKURA[44]	Albuterol 90µg pMDI two puffs PRN	[20,21]

Given that US clinical guidelines generally recommend two puffs of SABA as needed, in contrast to the protocol followed in some studies that utilized only one puff of terbutaline or salbutamol for reliever use, we adjusted the reported SABA actuation frequencies by doubling them to better align with US inhaler use patterns.

Abbreviations used: AHEAD, Comparison of the Efficacy/Safety of Symbicort® Turbuhaler®,Seretide™ Diskus™ 50/500 µg & Terbutaline Turbuhaler® 0.4 mg; Bec-Form, beclomethasone-formoterol; BID, twice daily; Bud-Form, budesonide-formoterol; COMPASS, Comparison versus higher fixed-dose of Symbicort and Seretide; DPI, dry powder inhaler; ED, emergency department; Flu-Sal, fluticasone-salmeterol; pMDI, pressurized metered-dose inhaler; PRN, as needed; SABA, short-acting beta-agonist; Salb, salbutamol; SAKURA, Study to Investigate the Efficacy of Symbicort® SMART; Terb, terbutaline; US, United States.

© 2026 Pham T et al. *JAMA Network Open.*

eReferences

1. Sobieraj DM, Weeda ER, Nguyen E, et al. Association of inhaled corticosteroids and long-acting β-agonists as controller and quick relief therapy with exacerbations and symptom control in persistent asthma: a systematic review and meta-analysis. *JAMA*. 2018;319(14):1485-1496. doi:10.1001/jama.2018.2769

2. Vogelmeier C, D'Urzo A, Pauwels R, et al. Budesonide/formoterol maintenance and reliever therapy: an effective asthma treatment option? *Eur Respir J*. 2005;26(5):819-828. doi:10.1183/09031936.05.00028305

3. Faissol DM, Griffin PM, Swann JL. Bias in Markov models of disease. *Math Biosci*. 2009;220(2):143-156. doi:10.1016/j.mbs.2009.05.005

4. Bentley TG, Kuntz KM, Ringel JS. Bias associated with failing to incorporate dependence on event history in Markov models. *Med Decis Making*. 2010;30(6):651-660. doi:10.1177/0272989X10363480

5. Weinstein M, O'Brien B, Hornberger J, et al. Principles of Good Practice for Decision Analytic Modeling in Health-Care Evaluation: Report of the ISPOR Task Force on Good Research Practices-Modeling Studies. *Value Health*. 2015;(2):19-28. doi:10.1046/j.1524-4733.2003.00234.x

6. Wickstrøm J, Dam N, Malmberg I, Hansen BB, Lange P. Cost-effectiveness of budesonide/formoterol for maintenance and reliever asthma therapy in Denmark–Cost-effectiveness analysis based on five randomised controlled trials. *Clin Respir J*. 2009;3(3):169-180. doi:10.1111/j.1752-699X.2009.00134.x

© 2026 Pham T et al. *JAMA Network Open.*

7. Arrotta N, Hill J, Villa-Roel C, Dennett E, Harries M, Rowe BH. Factors associated with hospital admission in adult patients with asthma exacerbations: A systematic review. *J Asthma*. 2019;56(1):34-41. doi:10.1080/02770903.2018.1424189

8. Agency for Healthcare Research and Quality. Medical Expenditure Panel Survey. Accessed October 1, 2024, https://meps.ahrq.gov/mepsweb/index.jsp

9. Liu J, Yu F, Song L. A systematic investigation on the research publications that have used the medical expenditure panel survey (MEPS) data through a bibliometrics approach. Library Hi Tech2020. p. 705-721.

10. U.S. Bureau of Labor Statistics. Medical care in U.S. city average, all urban consumers, not seasonally adjusted. 2024. Accessed November 1, 2024. Available from: https://data.bls.gov/timeseries/CUUR0000SAM?output_view=data. 2024.

11. Sanders GD, Neumann PJ, Basu A, et al. Recommendations for conduct, methodological practices, and reporting of cost-effectiveness analyses: second panel on cost-effectiveness in health and medicine. *JAMA*. 2016;316(10):1093-1103. doi:10.1001/jama.2016.12195

12. Levy J, Rosenberg M, Vanness D. A transparent and consistent approach to assess US outpatient drug costs for use in cost-effectiveness analyses. *Value Health*. 2018;21(6):677-684. doi:10.1016/j.jval.2017.06.013

13. Micromedex. Red Book. Accessed October 22, 2024, https://www.micromedexsolutions.com/

14. GoodRx. GoodRx. Accessed October 22, 2024, https://www.goodrx.com/

© 2026 Pham T et al. *JAMA Network Open.*

15. Somers J, Cook A. Prescription drug pricing in the private sector. A CBO Study Washington, DC: US Conggressional Budget Office2007.

16. Willke RJ. Beyond AWP... Way Beyond. *Value Health*. 2010;13(1)doi:10.1111/j.1524-4733.2009.00673.x

17. GoodRx. What is GoodRx? How does it work? Accessed October 24, 2024, https://support.goodrx.com/hc/en-us/articles/115004944026-What-is-GoodRx-How-does-it-work

18. Patel M, Pilcher J, Pritchard A, et al. Efficacy and safety of maintenance and reliever combination budesonide–formoterol inhaler in patients with asthma at risk of severe exacerbations: a randomised controlled trial. *Lancet Respir Med*. 2013;1(1):32-42. doi:10.1016/S2213-2600(13)70007-9

19. American Lung Association. Comparative Doses. Accessed October 22, 2024, https://www.lung.org/getmedia/9dc08936-a7e5-4796-9e9f-c0ffc8f6e768/Comparative-Doses-Chart.pdf

20. Johnson DB MB, Bounds CG. Albuterol. 2024. Accessed Jan 13, 2025. Available from: https://www.ncbi.nlm.nih.gov/books/NBK482272/. In: StatPearls [Internet]. Treasure Island (FL): StatPearls Publishing2024.

21. Chandra P, Paliwal L, Lodha R, Kabra S. Comparison of terbutaline and salbutamol inhalation in children with mild or moderate acute exacerbation of asthma. *Indian J Pediatr*. 2004;71:961-963. doi:10.1007/BF02828104

22. Kirsten A-M, Watz H, Brindicci C, Piccinno A, Magnussen H. Effects of beclomethason/formoterol and budesonide/formoterol fixed combinations on lung function and airway inflammation in patients with mild to moderate asthma–an

exploratory study. *Pulm Pharmacol Ther*. 2015;31:79-84. doi:10.1016/j.pupt.2014.08.007

23. Virchow JC, Rodriguez-Roisin R, Papi A, Shah TP, Gopalan G. A randomized, double-blinded, double-dummy efficacy and safety study of budesonide–formoterol Spiromax® compared to budesonide–formoterol Turbuhaler® in adults and adolescents with persistent asthma. *BMC Pulm Med*. 2016;16:1-11. doi:10.1186/s12890-016-0200-x

24. Weisfeld L, Shu Y, Shah TP. Bioequivalence of budesonide plus formoterol (BF) Spiromax® and BF Turbohaler®(with and without charcoal block) in healthy volunteers. *Int J Clin Pharmacol Ther*. 2015;53(7):593-602. doi:10.5414/CP202238

25. Bateman E, Silins V, Bogolubov M. Clinical equivalence of salmeterol/fluticasone propionate in combination (50/100 µg twice daily) when administered via a chlorofluorocarbon-free metered dose inhaler or dry powder inhaler to patients with mild-to-moderate asthma. *Respir Med*. 2001;95(2):136-146. doi:10.1053/rmed.2000.1008

26. Van Noord J, Lill H, Diaz TC, Greefhorst A, Davies P. Clinical equivalence of a salmeterol/fluticasone propionate combination product (50/500µg) delivered via a chlorofluorocarbon-free metered-dose inhaler with the Diskus™ in patients with moderate to severe asthma. *Clinical Drug Investigation*. 2001;21:243-255. doi:10.2165/00044011-200121040-00002

27. Brocklebank D, Ram F, Wright J, et al. Comparison of the effectiveness of inhaler devices in asthma and chronic obstructive airways disease: a systematic review of the literature. *Health Technol Assess*. 2001;5(26):22. doi:10.3310/hta5260

© 2026 Pham T et al. *JAMA Network Open.*

28. Morice AH, Ekelund J, Thorén A, Puterman AS. Comparable long-term safety and efficacy of a novel budesonide/formoterol pressurized metered-dose inhaler versus budesonide/formoterol Turbuhaler® in adolescents and adults with asthma. *Pulm Pharmacol Ther*. 2008;21(1):32-39. doi:10.1016/j.pupt.2006.10.006

29. Oh B-C, Lee J-E, Nam JH, Hong J-Y, Kwon S-H, Lee E-K. Health-related quality of life in adult patients with asthma according to asthma control and severity: A systematic review and meta-analysis. *Front Pharmacol*. 2022;13:908837. doi:10.3389/fphar.2022.908837

30. Sullivan PW, Ghushchyan VH, Campbell JD, Globe G, Bender B, Magid DJ. Measurement of utility in asthma: evidence indicating that generic instruments may miss clinically important changes. *Quality of Life Research*. 2016;25:3017-3026. doi:10.1007/s11136-016-1357-8

31. Tsuchiya A, Brazier J, McColl E, Parkin D. Deriving preference-based single indices from non-preference based condition-specific instruments: Converting AQLQ into EQ5D indices. MPRA Paper 29740, University Library of Munich, Germany2002.

32. Kuna P, Peters M, Manjra A, et al. Effect of budesonide/formoterol maintenance and reliever therapy on asthma exacerbations. *Int J Clin Pract*. 2007;61(5):725-736. doi:10.1111/j.1742-1241.2007.01338.x

33. Wilson SR, Rand CS, Cabana MD, et al. Asthma outcomes: quality of life. *J Allergy Clin Immunol*. 2012;129(3):S88-S123. doi:10.1016/j.jaci.2011.12.988

34. Bousquet J, Boulet L-P, Peters MJ, et al. Budesonide/formoterol for maintenance and relief in uncontrolled asthma vs. high-dose salmeterol/fluticasone. *Respir Med*. 2007;101(12):2437-2446. doi:10.1016/j.rmed.2007.07.014

© 2026 Pham T et al. *JAMA Network Open.*

35. Papi A, Corradi M, Pigeon-Francisco C, et al. Beclometasone–formoterol as maintenance and reliever treatment in patients with asthma: a double-blind, randomised controlled trial. *Lancet Respir Med*. 2013;1(1):23-31. doi:10.1016/S2213-2600(13)70012-2

36. Rabe KF, Atienza T, Magyar P, Larsson P, Jorup C, Lalloo UG. Effect of budesonide in combination with formoterol for reliever therapy in asthma exacerbations: a randomised controlled, double-blind study. *Lancet*. 2006;368(9537):744-753. doi:10.1016/S0140-6736(06)69284-2

37. Lloyd A, Price D, Brown R. The impact of asthma exacerbations on health-related quality of life in moderate to severe asthma patients in the UK. *Prim Care Respir J*. 2007;16(1):22-27. doi:10.3132/pcrj.2007.00002

38. Tan LE, Tan WHG, Aziz MIA, et al. Assessing the cost-effectiveness of mepolizumab as add-on therapy to standard of care for severe eosinophilic asthma in Singapore. *J Asthma*. 2022;59(1):189-199. doi:10.1080/02770903.2020.1837158

39. Sadatsafavi M, FitzGerald JM, O'Byrne PM, et al. The cost-effectiveness of as-needed budesonide-formoterol versus low-dose inhaled corticosteroid maintenance therapy in patients with mild asthma in Canada. *Allergy Asthma Clin Immunol*. 2021;17:1-13. doi:10.1186/s13223-021-00610-w

40. Ho JK, Shaker M, Greenhawt M, et al. Cost-effectiveness of budesonide-formoterol vs inhaled epinephrine in US adults with mild asthma. *Ann Allergy Asthma Immunol*. 2024;132(2):229-239. e3. doi:10.1016/j.anai.2023.10.024

© 2026 Pham T et al. *JAMA Network Open.*

41. Buendia JA, Guerrero Patiño D, Cossio-Giraldo YE. Cost-effectiveness of tiotropium versus omalizumab for uncontrolled allergic asthma. *J Asthma*. 2022;59(10):2016-2023. doi:10.1080/02770903.2021.1984527

42. Antonio Buendía J, Patiño DG. Cost-utility analysis of dupilumab add on therapy versus standard therapy in adolescents and adults for severe asthma in Colombia. *Expert Rev Pharmacoecon Outcomes Res*. 2022;22(4):575-580. doi:10.1080/14737167.2022.2011217

43. Zhou K, Zhang M, Zuo C, Xie X, Xuan J. Cost-effectiveness analysis of budesonide/formoterol SMART therapy versus salmeterol/fluticasone plus as-needed SABA among patients≥ 12 years with moderate asthma from the Chinese societal perspective. *J Med Econ*. 2024;27(1):1018-1026. doi:10.1080/13696998.2024.2385191

44. Atienza T, Aquino T, Fernandez M, et al. Budesonide/formoterol maintenance and reliever therapy via T urbuhaler versus fixed-dose budesonide/formoterol plus terbutaline in patients with asthma: Phase III study results. *Respirology*. 2013;18(2):354-363. doi:10.1111/resp.12009

45. Beasley R, Harrison T, Peterson S, et al. Evaluation of budesonide-formoterol for maintenance and reliever therapy among patients with poorly controlled asthma: a systematic review and meta-analysis. *JAMA Netw Open*. 2022;5(3):e220615-e220615. doi:10.1001/jamanetworkopen.2022.0615

46. Ställberg B, Naya I, Ekelund J, Eckerwall G. Real-life use of budesonide/formoterol in clinical practice: a 12-month follow-up assessment in a multi-national study of asthma patients established on single-inhaler maintenance and reliever therapy. *Int J Clin Pharmacol Ther*. 2015;53(6):447. doi:10.5414/CP202224

© 2026 Pham T et al. *JAMA Network Open.*

47. National Center for Health Statistics. NHIS Adult Summary Health Statistics. https://data.cdc.gov/d/25m4-6qqq

48. National Center for Health Statistics. NHIS Child Summary Health Statistics. Accessed November 15, 2025, https://data.cdc.gov/d/wxz7-ekz9

49. Song HJ, Blake KV, Wilson DL, Winterstein AG, Park H. Health-Related Quality of Life and Health Utilities of Mild, Moderate, and Severe Asthma: Evidence from the Medical Expenditure Panel Survey. *J Asthma Allergy*. 2021;14:929-941. doi:10.2147/jaa.S316278

50. Zureik M, Neukirch C, Leynaert B, Liard R, Bousquet J, Neukirch F. Sensitisation to airborne moulds and severity of asthma: cross sectional study from European Community respiratory health survey. *Bmj*. 2002;325(7361):411-4. doi:10.1136/bmj.325.7361.411

51. Braido F, Blasi F, Canonica GW, et al. Mild/Moderate Asthma Network in Italy (MANI): a long-term observational study. *J Asthma*. 2022;59(9):1908-1913. doi:10.1080/02770903.2021.1968895

52. Brusselle GG, Koppelman GH. Biologic Therapies for Severe Asthma. *N Engl J Med*. 2022;386(2):157-171. doi:10.1056/NEJMra2032506

53. Antonicelli L, Bucca C, Neri M, et al. Asthma severity and medical resource utilisation. *Eur Respir J*. 2004;23(5):723-9. doi:10.1183/09031936.04.00004904

54. Zaeh SE, Zimmerman ZE, Eakin MN, Chupp G. Adoption and implementation of maintenance and reliever therapy for adults with moderate-to-severe asthma. *Ann Allergy Asthma Immunol*. 2024;133(3):318-324. doi:10.1016/j.anai.2024.06.011

© 2026 Pham T et al. *JAMA Network Open.*

55. Rodríguez I, López-Caro JC, Gonzalez-Carranza S, et al. Adherence to inhaled corticosteroids in patients with asthma prior to and during the COVID-19 pandemic. *Sci Rep*. 2023;13(1):13086. doi:10.1038/s41598-023-40213-6

56. Bårnes CB, Ulrik CS. Asthma and adherence to inhaled corticosteroids: current status and future perspectives. *Respir Care*. 2015;60(3):455-68. doi:10.4187/respcare.03200

57. Stanford RH, Averell CM, Johnson PT, Buysman EK, Carlyle MH. Adherence and usage patterns of inhaled corticosteroids-long-acting beta-agonists by using inhaler-monitoring technology. *Allergy Asthma Proc*. 2020;41(4):256-264. doi:10.2500/aap.2020.41.200037

58. Parimi M, Svedsater H, Ann Q, et al. Persistence and Adherence to ICS/LABA Drugs in UK Patients with Asthma: A Retrospective New-User Cohort Study. *Adv Ther*. 2020;37(6):2916-2931. doi:10.1007/s12325-020-01344-8

© 2026 Pham T et al. *JAMA Network Open.*
