# Supplementary material for: Costs of Single Maintenance and Reliever Therapy vs Traditional Therapy for Asthma
Source: JAMA Netw Open. 2026 Feb 2;9(2):e2556757. doi: 10.1001/jamanetworkopen.2025.56757 (PMC12865660; doi:10.1001/jamanetworkopen.2025.56757)
Supplement: Supplement 2. — Data Sharing Statement [file jamanetwopen-e2556757-s002.pdf]

Data Sharing Statement

Pham. Costs of Single Maintenance and Reliever Therapy vs Traditional Therapy for Asthma. *JAMA Netw Open*. Published January 30, 2026. doi:10.1001/jamanetworkopen.2025.56757

Data
Data available: Yes
Data types: Other (please specify)
Additional Information: All analytical code and data input.
How to access data: Available upon email request to corresponding author.
When available: With publication

Supporting Documents
Document types: Statistical/analytic code
How to access documents: Kringsj@wustl.edu
When available: With publication

Additional Information
Who can access the data: Anyone requesting the data.
Types of analyses: For any purpose.
Mechanisms of data availability: Via email correspondence.
